# Nodal and *churchill1* position the expression of a notch ligand during *Xenopus* germ layer segregation

**DOI:** 10.26508/lsa.202201693

**Published:** 2022-09-30

**Authors:** María Belén Favarolo, Diego R Revinski, Matías J Garavaglia, Silvia L López

**Affiliations:** 1 Universidad de Buenos Aires, Facultad de Medicina, Departamento de Biología Celular e Histología/1° U.A. Departamento de Histología, Embriología, Biología Celular y Genética, Buenos Aires, Argentina; 2 CONICET–Universidad de Buenos Aires, Instituto de Biología Celular y Neurociencia “Prof. E. De Robertis” (IBCN), Laboratorio de Embriología Molecular “Prof. Dr. Andrés E. Carrasco”, Buenos Aires, Argentina; 3 Laboratorio de Bioinsumos, Instituto de Biotecnología, Universidad Nacional de Hurlingham, Buenos Aires, Argentina

## Abstract

In vertebrates, Nodal signaling plays a major role in endomesoderm induction, but germ layer delimitation is poorly understood. In avian embryos, the neural/mesoderm boundary is controlled by the transcription factor CHURCHILL1, presumably through the repressor ZEB2, but there is scarce knowledge about its role in other vertebrates. During amphibian gastrulation, Delta/Notch signaling refines germ layer boundaries in the marginal zone, but it is unknown the place this pathway occupies in the network comprising Churchill1 and Nodal. Here, we show that *Xenopus churchill1* is expressed in the presumptive neuroectoderm at mid-blastula transition and during gastrulation, upregulates *zeb2*, prevents *dll1* expression in the neuroectoderm, and favors neuroectoderm over endomesoderm development. Nodal signaling prevents *dll1* expression in the endoderm but induces it in the presumptive mesoderm, from where it activates Notch1 and its target gene *hes4* in the non-involuting marginal zone. We propose a model where Nodal and Churchill1 position Dll1/Notch1/Hes4 domains in the marginal zone, ensuring the delimitation between mesoderm and neuroectoderm.

## Abstract (precis)

Churchill and Nodal signaling, which participate in vertebrates’ germ layer induction, position a domain of Delta/Notch activity, which refines germ layer boundaries during frog gastrulation.

## Graphical Abstract

**Figure:**
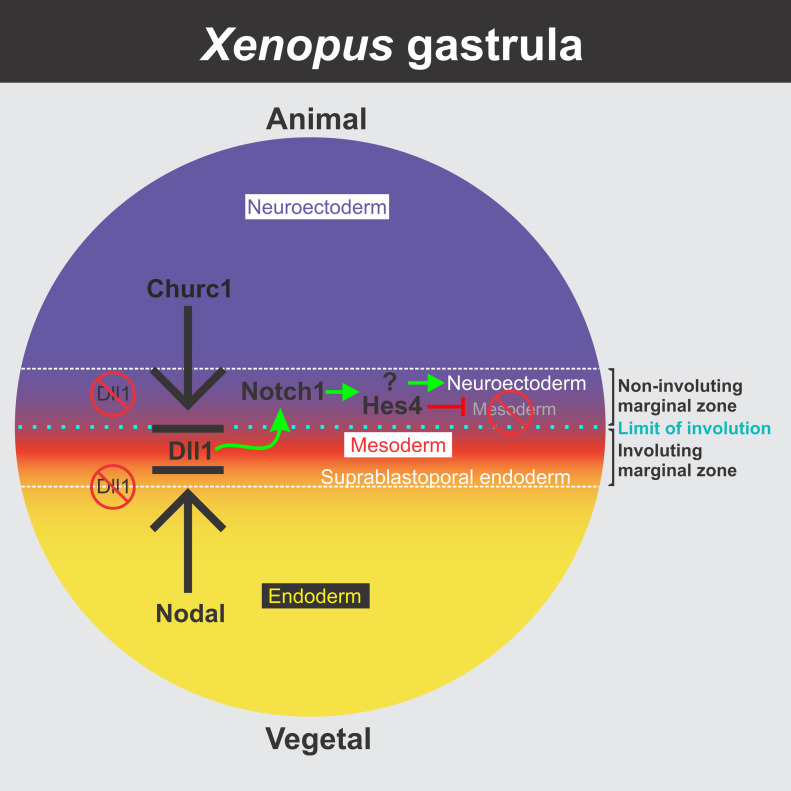

## Introduction

Forming the three germ layers (ectoderm, mesoderm, and endoderm), which give rise to all body tissues, is one of the first steps in diversifying pluripotent cells in vertebrates (Gilbert, 2014). They segregate during gastrulation, which drives endomesoderm internalization through morphogenetic movements typical for each organism (Keller et al, 2003; Stower & Bertocchini, 2017).

*Xenopus* has historically provided essential knowledge about vertebrates germ layer development. Their pre-gastrula arrangement in this amphibian model can be predicted along the egg’s Animal-Vegetal (An-Veg) axis. The ectoderm and sub-blastoporal endoderm derive from the animal and vegetal hemispheres, respectively. The equatorial region or marginal zone (MZ) mainly gives rise to mesoderm but also significantly contributes to ectoderm and supra-blastoporal endoderm derivatives (Dale & Slack, 1987; Moody, 1987). In the early gastrula, the MZ consists of two concentrically arranged rings surrounding the blastopore. The involuting MZ (IMZ) animally surrounds the blastopore and contains endomesoderm precursors being internalized (Keller & Danilchik, 1988; Shook et al, 2004). The non-involuting MZ (NIMZ), composed of presumptive ectodermal cells, animally surrounds the IMZ. It progressively converges and extends, occupying the space left on the surface by the IMZ because of its internalization, ultimately forming the blastopore margin at the end of gastrulation (Keller & Danilchik, 1988). Thus, the MZ is a transition area between germ layers, where their boundaries need to be defined for a correct allocation of mesodermal, endodermal, and ectodermal cells during gastrulation. While germ layer induction and specification were thoroughly studied in *Xenopus* (Kiecker et al, 2016; Charney et al, 2017b), how their boundaries are established and refined during their segregation is poorly understood.

In vertebrates, Nodal members of the TGFβ superfamily of secreted proteins represent the major endomesoderm inducers (Kiecker et al, 2016). TGFβs signal through type I and II receptors, which behave as serine/threonine kinases. Upon ligand binding, the type II receptor activates the type I receptor through phosphorylation. The latter phosphorylates R-Smad proteins (Smad2 and 3 in the Nodal pathway), which in turn, bind Smad4. Upon nuclear translocation, the complex interacts with DNA-specific binding proteins (like FoxH1 in the Nodal pathway), recruiting context-dependent co-activators or co-repressors to regulate transcription (Hill, 2001; Weiss & Attisano, 2013). In *Xenopus*, genes encoding Nodal1/2-6 are activated during two sequential waves of initial zygotic transcription (Takahashi et al, 2000; Collart et al, 2014). At the 256-cell stage, maternal VegT (present from oogenesis in the vegetal hemisphere) triggers *nodal5/6* transcription in the presumptive endoderm, which is dorsally enhanced by β-Catenin activity (Zhang & King, 1996; Takahashi et al, 2000; Skirkanich et al, 2011). An autoregulatory loop then reinforces *nodal5/6* activity (Skirkanich et al, 2011), which induces *nodal1/2/4* in the presumptive mesoderm (Takahashi et al, 2000). The transiently stronger Nodal cascade on the dorsal side contributes to inducing the gastrula organizer (GO) (Joseph & Melton, 1997; Agius et al, 2000; Takahashi et al, 2000; Schohl & Fagotto, 2002; Steiner et al, 2006; Zamparini et al, 2006; Kiecker et al, 2016; Reid et al, 2016). Nodal1/2-6 proteins are necessary for entire endomesoderm specification and patterning (Kiecker et al, 2016). Knockdown studies suggested that *nodal5/6* are dedicated to the endomesoderm program, whereas *nodal1/2* mainly control effectors of gastrulation movements, with a minor role in endomesoderm specification (Luxardi et al, 2010).

FGFs contribute to mesodermal induction and patterning in the early *Xenopus* embryo. Rather than true inducers, they are considered competence factors which allow mesodermal induction in response to TGFβ signaling (Kiecker et al, 2016) and also are required for neural induction by BMP antagonists (Stern, 2005). In avian embryos, FGF activity initially is involved in mesoderm induction but later promotes neural induction through the slow, indirect transcriptional activation of *CHURCHILL1* (*CHURC1*), which encodes a zinc finger transcriptional activator (Sheng et al, 2003). At the onset of chick gastrulation, *CHURC1* already is expressed in presumptive neural cells, preventing the activation of key mesodermal genes (such as *TBXT* and *TBX6*) and blocking cell ingression through the primitive streak by activating *ZEB2* (*zinc finger E-box binding homeobox 2*; previously known as *SIP1*), which encodes a homeodomain/zinc finger transcriptional repressor. *CHURC1* was proposed to control fate decision between neural and paraxial mesoderm, favoring neural development because cells that remain in the epiblast can receive neuralizing signals from the chicken GO (Sheng et al, 2003).

*churc1* is conserved in *Xenopus* (Sheng et al, 2003), but its spatial expression pattern during germ layer induction, specification, and segregation has not been described. RT-PCR assays revealed the presence of *churc1* transcripts at mid-gastrula (NF11.5), when it is positively regulated by the transcription factor Pou5f3.1 (formerly known as Pou91), which controls tissue competence during the transition from mesodermal to neural induction (Snir et al, 2006). In *Xenopus* animal caps, the induction of the pan-mesodermal marker *tbxt* by eFGF was blocked by *churc1* overexpression or *churc1VP16* mRNA (which encodes a chimeric *Xenopus* Churc1 protein fused to the VP16 transcriptional activating domain) but not by the dominant negative construct *churc1EnR* (encoding a chimeric *Xenopus* Churc1 protein fused to the Engrailed transcriptional repressor domain). In whole embryos, *tbxt* expression was suppressed from the IMZ by *churc1VP16* but not by *churc1EnR*. These results indicated that Churc1 behaves as a transcriptional activator and that the mechanism limiting avian mesoderm specification involving Churc1 is conserved in *Xenopus* (Sheng et al, 2003). However, markers of the other germ layers were not analyzed in frog embryos, and it was unclear if *churc1EnR* could actually up-regulate *tbxt*, which would more strongly support the hypothesis that normally, *churc1* restricts *tbxt* expression. Therefore, additional experiments, including a knockdown approach, were required in *Xenopus* to demonstrate this hypothesis. Moreover, it was not studied before if *churc1* regulates *zeb2* expression in *Xenopus*.

Notch signaling is typically initiated by interactions between neighboring cells, where the sending cell presents a transmembrane ligand belonging to the Delta and Jagged family (Dll/Jag). Once the ligand interacts with the transmembrane receptor Notch in the receiving cell, successive enzymatic cleavages release the Notch intracellular domain (NICD). Upon nuclear translocation, NICD forms a complex with the sequence-specific DNA-binding protein RBPJ, which recruits co-activators and activates Notch target genes (Bray, 2016). We have previously proposed that Dll1/Notch1 signaling is involved in neuroectoderm segregation from endomesoderm by refining germ layer boundaries in the MZ during gastrulation. Pre-involuted mesodermal cells of the IMZ present the Dll1 ligand to their neighbors on the other side of the limit of involution, thus preventing them to adopt the same fate (mesoderm). This is achieved by triggering the Notch pathway, which promotes neuroectoderm over mesoderm specification in the receiving cells, thus refining the limit of involution (Revinski et al, 2010).

Many genes of the HES1-7 group encoding bHLH-Orange transcriptional repressors are typical targets of the Notch/RBPJ pathway (Davis & Turner, 2001; Zhou et al, 2012). Amongst them, *hes4* is a good candidate for controlling the limit of involution position for several reasons (López, 2022). During gastrulation, *hes4* is expressed in scattered cells as a continuous ring throughout the NIMZ, complementing the pan-mesodermal marker *tbxt* (Aguirre et al, 2013). *hes4* overexpression repressed *tbxt* and blocked MZ cells involution (López et al, 2005). Upon *hes4* knockdown, the *tbxt* domain invaded the NIMZ territory, indicating that *hes4* controls the ectoderm/mesoderm boundary (Aguirre et al, 2013). Notch signaling is necessary and sufficient to activate *hes4* in different contexts in *Xenopus* (Glavic et al, 2004; López et al, 2005; Vega-López et al, 2015). Moreover, there is evidence that the *hes4* genomic locus has direct Notch/RBPJ responsiveness (Davis et al, 2001; Sakano et al, 2010). We have previously shown that the most conspicuous expression of *hes4* in the NIMZ is found in the dorsal-most part, marking prospective floor plate precursors in the GO region. Within this population, a bipotential switch controlled by *dll1/notch1* activates *hes4*, promoting floor plate over notochord fates (López et al, 2005). However, it was not addressed before if *hes4* is regulated by *notch1/dll1* throughout the remainder of the NIMZ, outside the GO.

Notably, the Notch pathway was not included in the last gene network proposed for endomesoderm formation in *Xenopus* (Charney et al, 2017b), despite the abundant evidence about its participation in germ layer development in bilaterians (Favarolo & López, 2018). Strikingly, Delta/Notch is a key signaling pathway for mesoderm or endoderm induction and specification in invertebrates. However, in vertebrates, Nodal signaling appears as the main player in endomesoderm induction, whereas Delta/Notch signaling rather seems to refine the limits between germ layers (Favarolo & López, 2018). Therefore, it was necessary to understand where the Delta/Notch pathway is placed in the gene network controlling germ layer development in vertebrates.

Given the importance of Nodal signaling in endomesoderm induction in vertebrates, CHURC1’s role in setting boundaries between endomesoderm and neuroectoderm in avian embryos, and the previous evidence that Dll1/Notch1 refines germ layer delimitation in vertebrates, we employed the *Xenopus* model to study if the Dll1/Notch pathway is controlled by *churc1* and Nodal signaling. To address this, we first analyzed the spatial expression pattern of *churc1* transcripts in *Xenopus*, which was previously unknown, showing their early presence in presumptive neural territories. We then performed a more detailed analysis of *churc1* role on germ layer development in this model and found that it is necessary to restrict endomesodermal fates and for neural development and *zeb2* expression. We then confirmed that Dll1/Notch1 signaling controls the position of the *hes4* domain throughout the NIMZ and found that Nodal signaling prevents *dll1* expression in the endoderm but induces it in the presumptive mesoderm, from where it activates Notch1 and *hes4* in the NIMZ. We propose a model where Nodal and Churchill1 position Dll1/Notch1/Hes4 domains in the MZ, ensuring the delimitation between mesoderm and neuroectoderm.

## Results

### Early expression pattern of *churc1*

**Figure 1.**
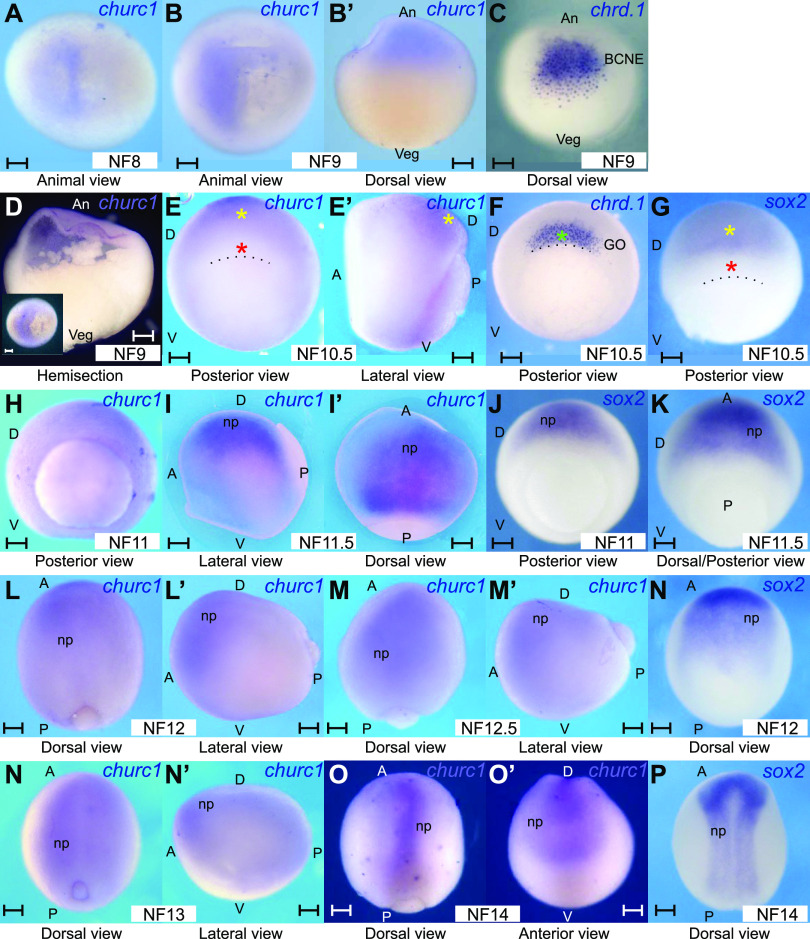
*churc1* mRNA distribution in *Xenopus laevis* embryos. **(A, B, B’, D, E, E’, H, I, I’, L, L’, M, M’, N, N’, O, O’)** Expression of *churc1* mRNA from mid-blastula to early neurula. **(C)** Expression of *chrd.1* mRNA at late blastula. **(F)** Expression of *chrd.1* mRNA at early gastrula. **(G, J, K, N, P)** Expression of *sox2* mRNA from early gastrula to early neurula. **(A)** Mid-blastula, animal view. **(B, B’, C, D)** Late blastula embryos, shown in animal view (B), dorsal view (B’, C), or bisected along the mid–sagittal plane (D). The inset in (D) shows the same embryo in animal view before being bisected. **(E, E’, F, G)** Early gastrula embryos in posterior (E, F, G) and lateral views (E’). **(H, I, I’ J, K)** Mid-gastrula embryos in posterior (H, J, K), lateral (I), and dorsal views (I’). **(L, L’, M, M’, N)** Late gastrula embryos in dorsal (L, M, N) and lateral views (L’, M’). **(N, N’, O, O’, P)** Embryos at the neural plate stage, shown in dorsal (N, O, P), lateral (N’), or anterior views (O’). All embryos were processed for in situ hybridization and photographed in PBS, except in (A, B, B’, D), which were photographed in 50% glycerol/PBS for better transparency. All images are from albino embryos, except (I, I’), which correspond to a bleached, wild-type embryo. The dotted line in (E, F, G) demarcates the dorsal blastopore lip. An, animal; Veg, vegetal; A, anterior; P, posterior; D, dorsal; V, ventral; BCNE, Blastula- Chordin- and Noggin-expressing center; GO, gastrula organizer; np, developing neural plate. NF, stages according to Nieuwkoop and Faber (1994). Yellow asterisks mark *churc1* and *sox2* expression in the presumptive neural plate. Red asterisks mark absence of *churc1* and *sox2* transcripts from the GO. The green asterisk marks *chrd.1* expression in the GO. Scale bars: 0.2 mm.

Since the spatial expression pattern of *churc1* in *Xenopus* was not previously described, we performed in situ hybridization (ISH) from mid-blastula to early neurula stages. At NF8, expression was restricted to one-half of the animal hemisphere (Fig 1A), persisting there at late blastula (Fig 1B, B’, and D), when *chordin.1* (*chrd.1*), which encodes a BMP antagonist and neural inducer, is readily expressed at the BCNE center (Kuroda et al, 2004; Castro Colabianchi et al, 2021) (Fig 1C). The BCNE comprises animal and marginal cells at the blastula’s dorsal region and contains precursors of the GO, forebrain, and most of the midbrain and hindbrain (Kuroda et al, 2004). At the onset of gastrulation, *churc1* transcripts are distributed like *sox2* mRNA (yellow asterisk, Fig 1E, E’, and G), which encodes an HMG-box transcription factor of the SoxB1 family, expressed by immature, undifferentiated neuroectodermal progenitors, revealing their commitment to a neural plate fate (Stern, 2006; Rogers et al, 2008). *churc1* and *sox2* transcripts are absent from the GO (red asterisks, Fig 1E and G), where *chrd.1* expression persists (green asterisk, Fig 1F), whereas the transient expression of this neural inducer in brain precursors previously found at the BCNE disappeared by this stage (Kuroda et al, 2004) (compare Fig 1C and F). *churc1* and *sox2* transcripts share a similar distribution until the last stage analyzed (neural plate) (Fig 1H–P). In conclusion, *churc1* expression begins in the presumptive neuroectoderm before the appearance of overt signs of neural induction and later persists in the developing neuroectoderm.

### *Churc1* disfavors IMZ lineages and is required for neural specification

A previous study of *churc1* role during *Xenopus* embryogenesis was limited to testing the effects of the activator Churc1VP16 and the repressor Churc1EnR constructs on the pan-mesoderm marker *tbxt* at gastrula stage (Sheng et al, 2003), but other germ layers were not analyzed. Moreover, knockdown experiments were not performed to validate *churc1* role in *Xenopus* germ layer development. We addressed these issues by unilaterally injecting embryos at the four-cell stage with the *Xenopus churc1EnR* and *churc1VP16* mRNAs previously employed by Sheng et al (2003), *churc1* mRNA, and a morpholino oligonucleotide designed to inhibit *churc1* translation (*churc1* MO) (this study). At gastrula stage, we examined the expression of the following markers: *sox2* (neuroectoderm) (Piccolo et al, 1997; Rogers et al, 2008), *sox17a* (endoderm) (Hudson et al, 1997), and *tbxt* (pan-mesoderm) (Smith et al, 1991).

**Figure 2.**
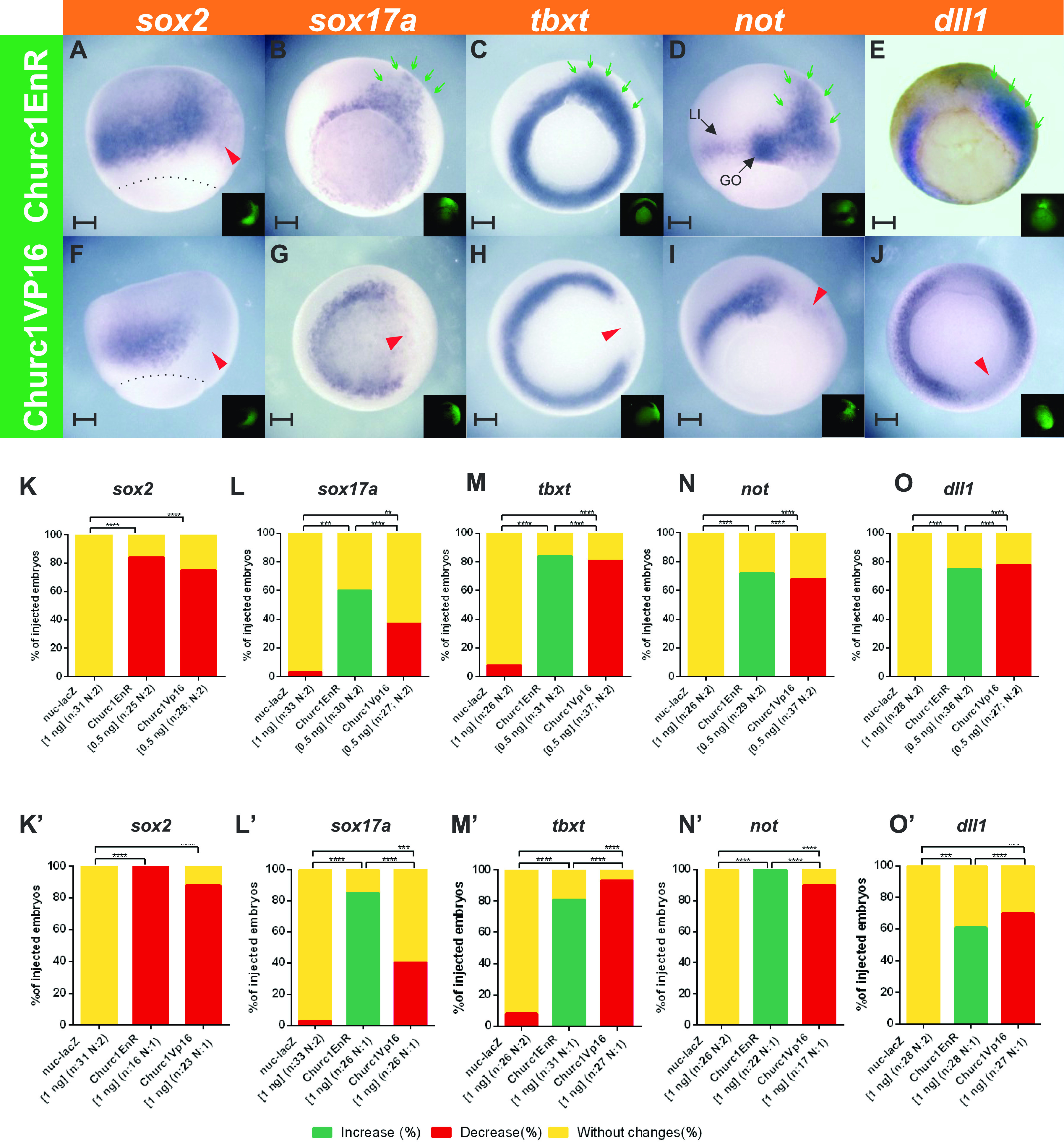
Effects of *Xenopus churc1* repressor and activator constructs on germ layer specification and *dll1* expression during gastrulation. Embryos were injected into one dorsal cell at the four-cell stage with: **(A, B, C, D, E)** *churc1EnR* mRNA. **(F, G, H, I, J)** *churc1VP16* mRNA. They were allowed to develop until the gastrula stage when they were analyzed through in situ hybridization for the following markers: (A, F) *sox2* (neuroectoderm). **(B, G)** *sox17a* (endoderm). **(C, H)** *tbxt* (pan-mesoderm). **(D, I)** *not* (GO, gastrula organizer; LI, limit of involution). **(E, J)** *dll1* (Notch ligand). All photographs are oriented with the injected side towards the right. The injected side was revealed by the green fluorescence of the dextran tracer (shown in the insets). Dotted lines delineate the blastopore. For each marker, the expression was compared between the injected- and the non-injected sides. Red arrowheads point to repression, and green arrows, to domain expansions on the injected side. Scale bars: 0.2 mm. **(K, L, M, N, O, K’, L’, M’, N’, O’)** Graphs comparing the effects between *churc1EnR* mRNA, *churc1VP16* mRNA, and *nuc-lacZ* mRNA (see Fig S1). Results are represented as the percentage of injected embryos showing increase (green), decrease (red), or no changes (yellow) in the expression of *sox2* (K, K’), *sox17a* (L, L’), *tbxt* (M, M’), *not* (N, N’), or *dll1* (O, O’) on the injected side in comparison to the non-injected side. **(K, L, M, N, O)** Comparison between 0.5 ng of *churc1EnR* mRNA, 0.5 ng of *churc1VP16* mRNA, and 1 ng of *nuc-lacZ* mRNA. **(K’, L’, M’, N’, O’)** Comparison between 1 ng of *churc1EnR* mRNA, 1 ng of *churc1VP16* mRNA, and 1 ng of *nuc-lacZ* mRNA. Asterisks indicate significant differences between treatments (Chi-square test; ****P < 0.0001; ***P = 0.0001; **P = 0.0032; n, total number of analyzed embryos for each marker; N, number of independent experiments).

**Figure S1.**
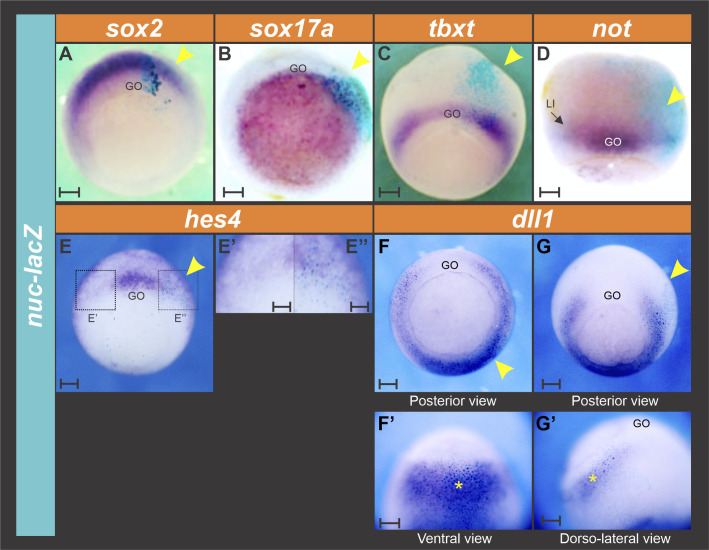
Injection of *nuc-lacZ* mRNA does not affect the expression domains of the markers analyzed in this work. **(A, B, C, D, E, E’, F, F’, G, G’)** One cell of four-cell stage embryos was injected with 1 ng (A, B, C, D, F, F’, G, G’) or 1.5 ng (E, E’) of *nuc-lacZ* mRNA as control. At gastrula stage, the expression of the following markers was analyzed through in situ hybridization: (A) *sox2* (neuroectoderm). **(B)** *sox17a* (endoderm). **(C)** *tbxt* (pan-mesoderm). **(D)** *not* (GO, gastrula organizer; LI, limit of involution). **(E, E’’)** *hes4*. **(F, F’,G, G’)** *dll1*. For each marker, expression was compared between the injected- (revealed by the enzymatic activity of β-galactosidase, turquoise staining) and the non-injected side. All photographs are oriented with the injected side towards the right. Yellow arrowheads and asterisks indicate that the expression of the analyzed marker was not changed on the injected side in comparison with the contralateral, non-injected side. **(E’, E’’)** Magnifications of the areas indicated by dotted squares in (E). **(F, F’)** and **(G, G’)** show two different embryos hybridized with *dll1*, one unilaterally injected on the ventral side (F, F’) and the other one, at the dorsal side (G, G’). Scale bars: 0.2 mm, except in (E’, E’’), where the scale bar represents 0.1 mm.

**Figure 3.**
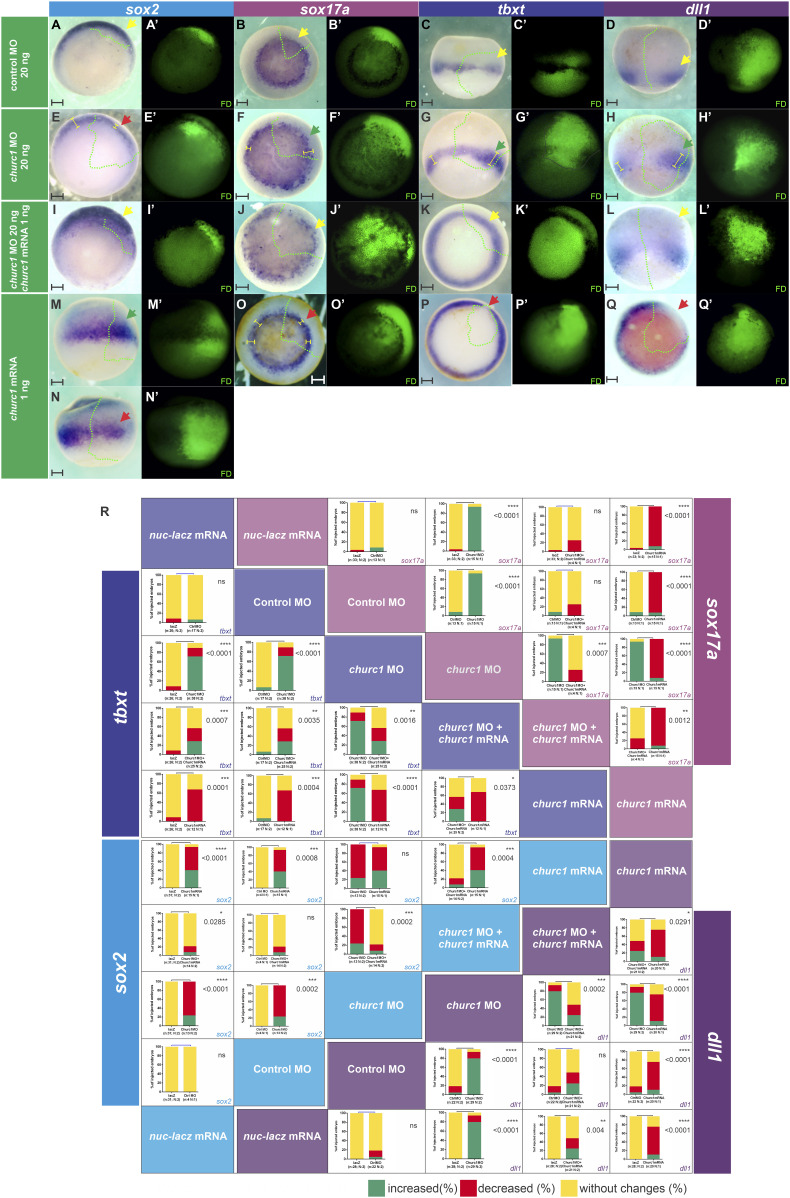
Effects of *Xenopus churc1* knockdown and *churc1* overexpression on germ layer specification and *dll1* expression during gastrulation. **(A, B, C, D)** Embryos were injected into one dorsal cell at the four-cell stage with the following molecules: (A, B, C, D) Control MO (20 ng). **(E, F, G, H)** *churc1* MO (20 ng). **(I, J, K, L)** *churc1* MO (20 ng) + *churc1* mRNA (1 ng). **(M, N, O, P, Q)** *churc1* mRNA (1 ng). **(A, E, I, M, N)** They were allowed to develop until gastrula stage when they were analyzed through in situ hybridization for the following markers: (A, E, I, M, N) *sox2* (neuroectoderm). **(B, F, J, O)** *sox17a* (endoderm). **(C, G, K, P)** *tbxt* (pan-mesoderm). **(D, H, L, Q)** *dll1* (Notch ligand). All photographs are oriented with the injected side towards the right. **(A’, B’, C’, D’, E’, F’, G’, H’, I’, J’, K’, L’, M’, N’, O’, P’, Q’)** Fluorescence microscopy images corresponding to the bright field images shown in (A, B, C, D, E, F, G, H, I, J, K, L, M, N, O, P, Q) revealing the green fluorescence of the dextran tracer (FD) which marks the injected side. The borders of the fluorescent regions are projected with green dotted lines into the corresponding bright field views in (A, B, C, D, E, F, G, H, I, J, K, L, M, N, O, P, Q). Yellow segments indicate the domain width for each marker. Red, green, and yellow arrows point to decreased, expanded, or unperturbed expression, respectively, of the analyzed marker on the injected side in comparison to the non-injected side. Scale bars: 0.2 mm. **(R)** Graphs comparing the effects between *nuc-lacZ* mRNA (1 ng), control MO (20 ng), *churc1* MO (20 ng), *churc1* MO (20 ng) + *churc1* mRNA (1 ng), and *churc1* mRNA (1 ng). Graphs represent the percentages of injected embryos showing increase (green), decrease (red), or no changes (yellow) for the expression domains of *tbxt* (triangular matrix labeled in blue), *sox2* (triangular matrix labeled in cyan), *sox17a* (triangular matrix labeled in purplish pink), and *dll1* (triangular matrix labeled in violet) on the injected side in comparison to the non-injected side. Representative images of *nuc-lacZ* mRNA-injected embryos are shown in Fig S1. Each triangular matrix represents the comparisons between injections (internal labeled blocks on the hypotenuse) for each marker (lateral labeled blocks). For example, to compare the *churc1* mRNA and the *lacZ* mRNA injections for the *dll1* marker, see the intersection between the *churc1* mRNA column and the *nuc-lacZ* mRNA row at the bottom left corner of the figure. n, total number of analyzed embryos. N, number of independent experiments. Asterisks indicate significant differences between treatments (*P* < 0.05; Chi-Square test). *P*-values are shown within each panel. ns, non-significant differences.

*Churc1EnR* noticeably suppressed *sox2* expression (Fig 2A, K, and K’), whereas the suprablastoporal endoderm and the involuting mesoderm were noticeably expanded, as revealed by *sox17a* (Fig 2B, L, and L’) and *tbxt* (Fig 2C, M, and M’), respectively. These changes were statistically significant in comparison to embryos unilaterally injected with *nuc-lacZ* mRNA, which essentially did not affect *sox2* (Figs 2K and K’ and S1A), *sox17a* (Figs 2L and L’ and S1B), or *tbxt* (Figs 2M and M’ and S1C). Knockdown with *churc1* MO produced similar results on the expression of germ layer markers to those obtained with *churc1EnR* (Fig 3E–G and R). Interestingly, since the MO effects were milder, rather than the complete suppression of *sox2* obtained with *churc1EnR*, we could appreciate a reduction of the *sox2* domain on the *churc1* MO-injected side (Fig 3E, E’, and R), whereas the *sox17a* and *tbxt* domains were complementary expanded over the neuroectoderm (Fig 3F–G’ and R). This animal shift of the neuroectoderm/endomesoderm boundary was significant in comparison to control MO unilateral injections, which essentially did not affect *sox2*, *sox17a* or *tbxt* on the injected side (Fig 3A–C’ and R) and was significantly rescued by co-injection of 1 ng of *churc1* mRNA (Fig 3I–K’ and R), confirming that the effects of *churc1* MO were specific. Since either *churc1EnR* mRNA or *churc1* MO expanded the endomesoderm, the great majority (84%) or all the embryos (100%) injected with 0.5 or 1 ng of *churc1EnR* mRNA, respectively, and the great majority (77%) of embryos injected with *churc1* MO showed a significant decrease of the *sox2* domain, our results suggest that *churc1* normally inhibits endomesoderm and is required for neuroectoderm development.

To verify if *churc1* can promote neuroectoderm development at the expense of the endomesoderm, gain of function experiments were performed with the *churc1* activating form (*churc1VP16*) or *churc1* overexpression. As expected, *churc1VP16* significantly suppressed *sox17a* and *tbxt* (Fig 2G, H, L, L’, M, and M’) and 1 ng of *churc1* mRNA significantly reduced the extent of their domains on the injected side (Fig 3O, P, and R), albeit these effects were milder than those obtained with *churc1VP16*. These results confirm that *churc1* disfavors endomesoderm development. Strikingly, rather than expanding the *sox2* domain, as expected if *churc1* favored neuroectoderm, the potent activating construct *churc1VP16* significantly suppressed *sox2* in the great majority of embryos (Fig 2F, K, and K’; 75% for 0.5 ng, 88% for 1 ng of *churc1VP16* mRNA). This result might be explained because mesoderm specification is strongly suppressed by this construct and therefore, embryos would lack the signals necessary for neural induction and stabilization of the neural fate emitted by the GO, its precursors, and its descendants (Stern, 2005, 2006; Stern et al, 2006). In fact, overexpressing 1 ng of *churc1* mRNA significantly changed *sox2* expression, producing two distinct phenotypes with similar frequencies: in 40% of the embryos the *sox2* domain was expanded (this was the expected phenotype if *churc1* favored neural specification) (Fig 3M, M’, and R), and in 53% of the embryos, *sox2* expression was reduced (Fig 3N, N’, and R). This second phenotype is similar to but milder than that obtained with *churc1VP16*, which strongly suppressed *sox2* in the great majority of cases (75% and 88% of embryos injected with 0.5 and 1 ng of *churc1VP16* mRNA, respectively). Since the effects of 1 ng of *churc1* mRNA are rather moderate in comparison to those obtained with 0.5 or 1 ng of the *churc1VP16* activating construct, we interpret that in those embryos with milder mesodermal defects it was still possible to observe neuroectoderm expansions, as expected according to the hypothesis that *churc1* favors neuroectoderm development.

**Figure 4.**
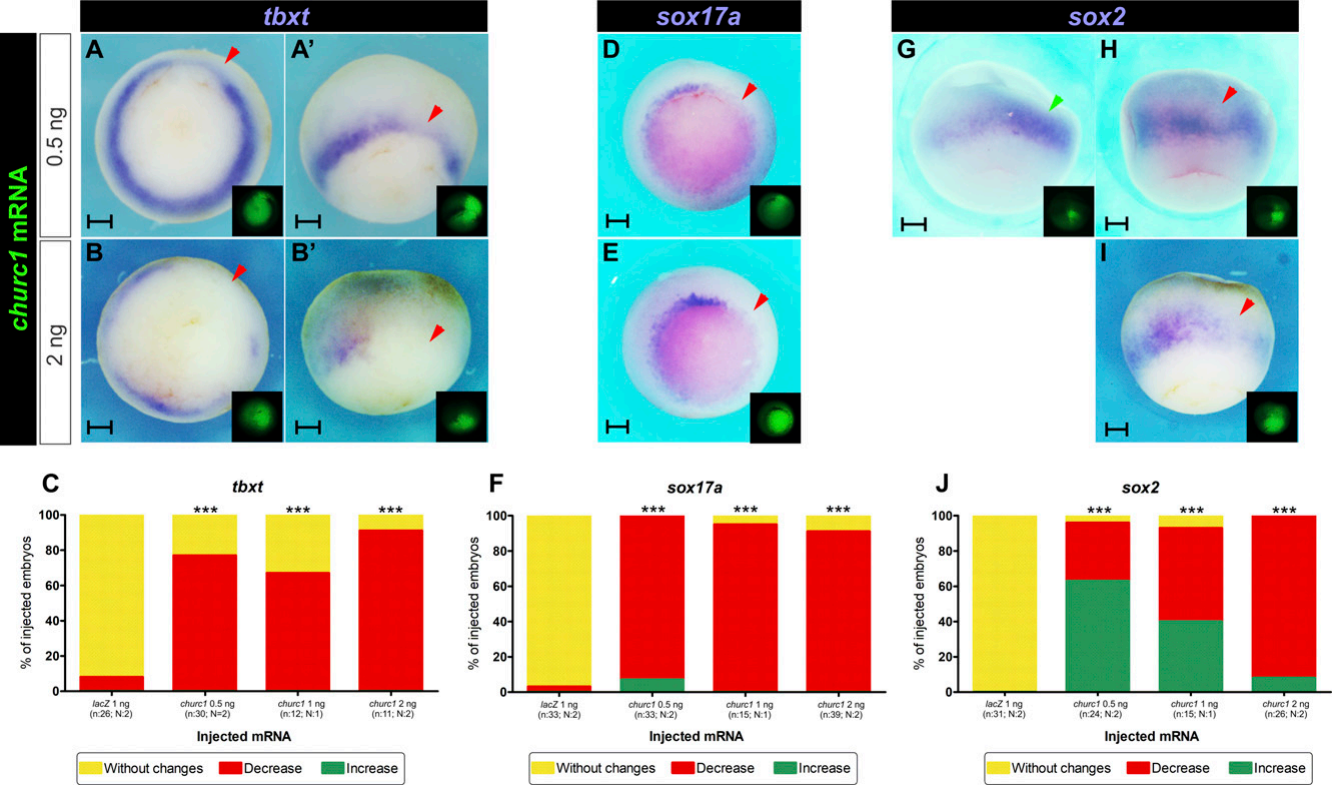
Effects of different doses of *churc1* mRNA overexpression on germ layers markers at gastrula stage. **(A, B, C, D, E, F, G, H, I)** Embryos injected into one dorsal cell at the four-cell stage with 0.5 ng of *churc1* mRNA (A, A’, D, G, H) or 2 ng of *churc1* mRNA (B, B’, E, I). They were allowed to develop until gastrula stage when they were analyzed through in situ hybridization for the following markers: (A, A’, B, B’) *tbxt* (pan-mesoderm). **(D, E)** *sox17a* (endoderm). **(G, H, I)** *sox2* (neuroectoderm). All photographs are oriented with the injected side towards the right. The injected side was revealed by the green fluorescence of the dextran tracer (FD, shown in the insets). For each marker, expression was compared between the injected- and the non-injected sides. Red and green arrowheads point to decreased or reduced expression, respectively, of the analyzed marker on the injected side. Embryos are shown in posterior (A, B, D, E) or dorsal views (A’, B’, G, H, I). The dorsal views shown in (A’, B’) are from the same embryos shown in posterior view in (A, B), respectively. Scale bars: 0.2 mm. **(C, F, J)** Graphs comparing the effects of 0.5, 1, and 2 ng of *churc1* mRNA, and 1 ng of *nuc-lacZ* mRNAs on the expression of *tbxt* (C), *sox17a* (F), and *sox2* (J). For each marker, results are represented as the percentage of injected embryos showing increase (green), decrease (red), or no changes (yellow) on the injected side in comparison to the non-injected side. Results from the injections of 1 ng of *nuc-lacZ* mRNA or 1 ng of *churc1* mRNA to build these graphs are shown in Figs S1 and 3, respectively. Asterisks indicate significant differences between *churc1* mRNA and *lacZ* mRNA injections (***P < 0.0001; Chi-Square test). n, total number of analyzed embryos. N, number of independent experiments.

**Figure 5.**
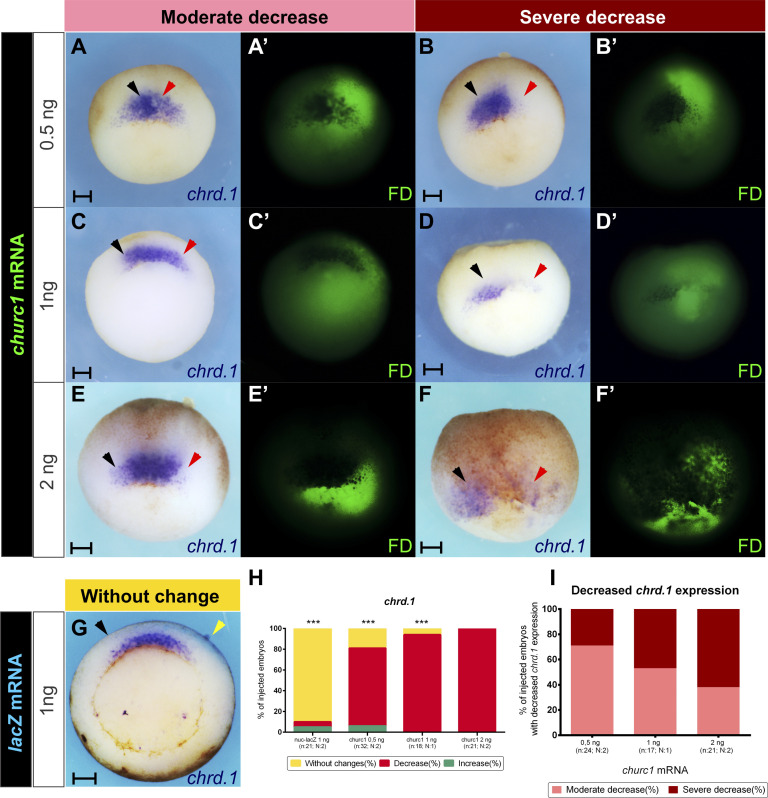
Effects of different doses of *churc1* mRNA overexpression on *chrd.1* expression at gastrula stage. **(A, B, C, D, E, F, G)** Expression of transcripts encoding the neural inducer Chrd.1 revealed by in situ hybridization at gastrula stage in embryos that were injected into one dorsal cell at the four-cell stage with the following mRNAs: (A, B) 0.5 ng of *churc1*. **(C, D)** 1 ng of *churc1*. **(E, F)** 2 ng of *churc1*. **(G)** 1 ng of *nuc*-*lacZ*. **(A’, B’, C’, D’, E’, F’)** Fluorescence microscopy images corresponding to the bright field images shown in (A, B, C, D, E, F), respectively, revealing the green fluorescence of the dextran tracer (FD) which marks the injected side. **(G)** The injected side in (G) was revealed by the enzymatic activity of β-galactosidase (turquoise staining). In all photographs, the injected side is oriented towards the right. Black arrowheads point to *chrd.1* expression on the non-injected side. Red and yellow arrowheads point to decreased or unperturbed *chrd.1* expression, respectively, on the injected side, in comparison to the non-injected side. Scale bars: 0.2 mm. **(H)** Graph comparing the effects on *chrd.1* expression of 0.5, 1, and 2 ng of *churc1* mRNA, and 1 ng of *nuc-lacZ* mRNA. Results are represented as the percentage of injected embryos showing increase (green), decrease (red), or no changes (yellow) of *chrd.1* expression on the injected side in comparison to the non-injected side. Asterisks indicate significant differences between *churc1* mRNA and *lacZ* mRNA injections (***P < 0.0001; Chi-Square test). **(I)** Graph representing the percentage of embryos with moderate (pink), as shown in (A, C, E), or severe decrease (wine red) of *chrd.1* expression, as shown in (B, D, F), after *churc1* mRNA overexpression, considering as 100% the total number of embryos showing *chrd.1* downregulation. n, total number of analyzed embryos. N, number of independent experiments.

To corroborate this, we compared the effects of overexpressing 0.5, 1, and 2 ng of *churc1* mRNA on the same germ layer markers and the neural inducer *chrd.1*, which is normally expressed in the GO (de Robertis & Kuroda, 2004). All tested doses of *churc1* mRNA significantly decreased *sox17a*, *tbxt*, and *chrd.1* expression when compared to *lacZ* mRNA injections (Figs 3–5 and S1), with more severe repressions at the highest dose. *churc1* mRNA also significantly perturbed *sox2* expression at all amounts tested (Figs 3–5 and S1). Remarkably, the increase of this neural marker prevailed with the lowest dose of *churc1* mRNA whilst *sox2* repression prevailed with the highest dose (Figs 3 and 4). Concomitantly, a moderate decrease in *chrd.1* expression prevailed with the lowest dose of *churc1* mRNA whilst a severe *chrd.1* repression prevailed with the highest dose (Fig 5). These results indicate that at lower doses, *churc1* mRNA overexpression favors neural development, most likely because sufficient levels of neural inducers like Chrd.1 are present to allow neural induction. In contrast, higher doses of *churc1* mRNA strongly repressed mesoderm development and neural inducers like Chrd.1 would not reach sufficient levels to promote neural induction, thus explaining the decrease of *sox2* expression.

**Figure S2.**
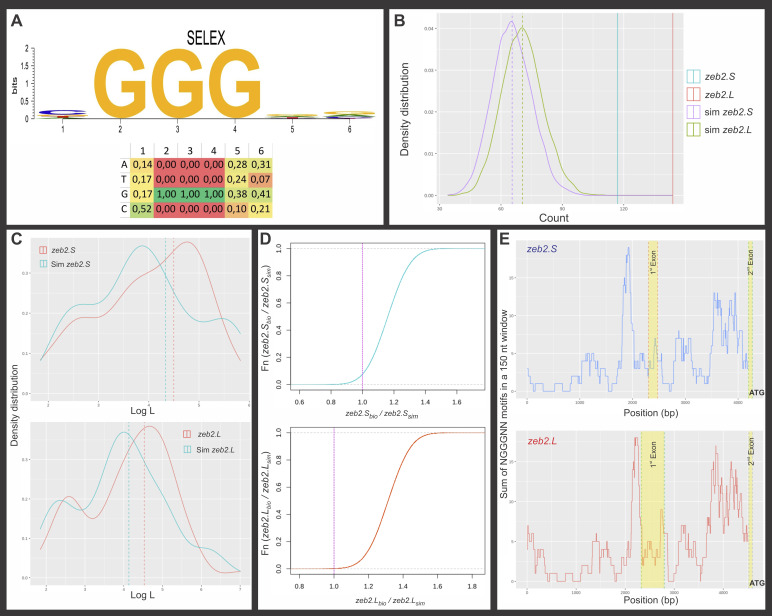
In-silico analysis of putative Churc1 binding sites in *Xenopus laevis zeb2* homeologs. **(A)** Sequence logo and frequency table describing the degree of information and the frequency per position of the Churc1-binding motif adapted from Sheng et al (2003). **(B)** Kernel density plot. Density distribution (N = 10,000, Bandwidth = 1.257) of the NGGGNN motifs present in the two collections of 10,000 simulated (sim) sequences with the same nucleotide composition and length of *X. laevis zeb2.S* and *zeb2.L* regions analyzed and in the corresponding biological genomic regions of both homeologs. Dashed lines represent the mean for each collection of simulated sequences ($\bar{x}$ = 65.45 ± 9.63 for sim *zeb2.S*; $\bar{x}$ = 70.47 ± 9.98 for sim *zeb2.L*). The vertical light blue line represents the 117 motifs present in *zeb2.S* biological sequence and the red one, the 144 motifs present in the *zeb2.L* biological sequence. **(C)** Density distribution of NGGGNN motifs for the biological *zeb2* sequences (red lines; 117 motifs for *zeb2.S*, 144 motifs for *zeb2.L*) and their simulated sequences (light blue lines; 117 motifs randomly selected for sim *zeb2.S* and 144 for sim *zeb2.L*; one example of the 1,000 Monte Carlo generated data is shown). Dashed lines: mode for each distribution. **(D)** Empirical Cumulative Density Function graphs showing the results of the 100,000 Monte Carlo simulations when the success rate of the *zeb2* biological sequences (bio) is divided by the success rates from simulated sequences (sim). The success rate of the biological sequence was higher than that of the simulated sequences in 90% and 99% of the 100,000 simulations for *zeb2.S* and *zeb2.L*, respectively. Pink dashed lines: threshold above which the success rate of the biological sequence is higher than that of the simulated sequences. **(E)** Distribution of putative Churc1 binding sites in a region containing the promoters of both *zeb2* homeologs. Yellow boxes: first exon and the beginning of the second exon, containing the translation start site (ATG). Two peaks of enrichment in NGGGNN motifs are immediately upstream of the first and second exons, coincident with human ZEB2 (Sheng et al, 2003).

We also analyzed the effects of *churc1EnR* and *churc1VP16* on *not* expression, which encodes a homeodomain transcription factor (von Dassow et al, 1993). During gastrulation, *not* is expressed in the GO (Figs 2D and S1D), marking the dorsal midline (DML) precursors that will later populate the notochord, the neural tube floor plate, and the endodermal DML (von Dassow et al, 1993). In addition, *not* is expressed in a ring with diffuse edges of positive scattered cells demarcating the transition border between neuroectoderm and endomesoderm during gastrulation, corresponding to the limit of involution (von Dassow et al, 1993) (Figs 2D and S1D). Therefore, we focused our attention on the limit of involution domain. While *nuc-lacZ* did not affect this domain on the injected side (Figs S1D and 2N and N’), *Churc1EnR* significantly expanded it towards the animal pole (Fig 2D, N, and N’), whereas the activating VP16 form significantly suppressed it (Fig 2I, N, and N’). Therefore, when Churc1-target genes were repressed, the transition border between germ layers was expanded, with a predominance of endomesodermal precursors (as revealed by the expansion of *tbxt* and *sox17a* in the same group of embryos) at the expense of the neuroectodermal fate (*sox2*).

Overall, our results support the hypothesis that in *Xenopus*, *churc1* controls the limit of involution position, disfavoring the development of IMZ cell lineages (mesoderm and endoderm) and favoring neural specification if neural inducers are present.

### *Churc1* restricts *dll1* expression to the IMZ

The effects produced by *churc1EnR* and *churc1* MO on germ layer markers were similar (although with stronger suppressions and expansions in the case of the repressor construct) to those previously obtained when the Notch pathway was blocked at the Dll1 ligand’s level (Revinski et al, 2010). This suggests that *churc1* and the Dll1/Notch pathway might be linked during germ layer development. Therefore, we wondered if *churc1* is capable of regulating the Dll1/Notch pathway and we examined *dll1* expression at gastrula stage after activating or blocking *churc1* function.

**Figure S3.**
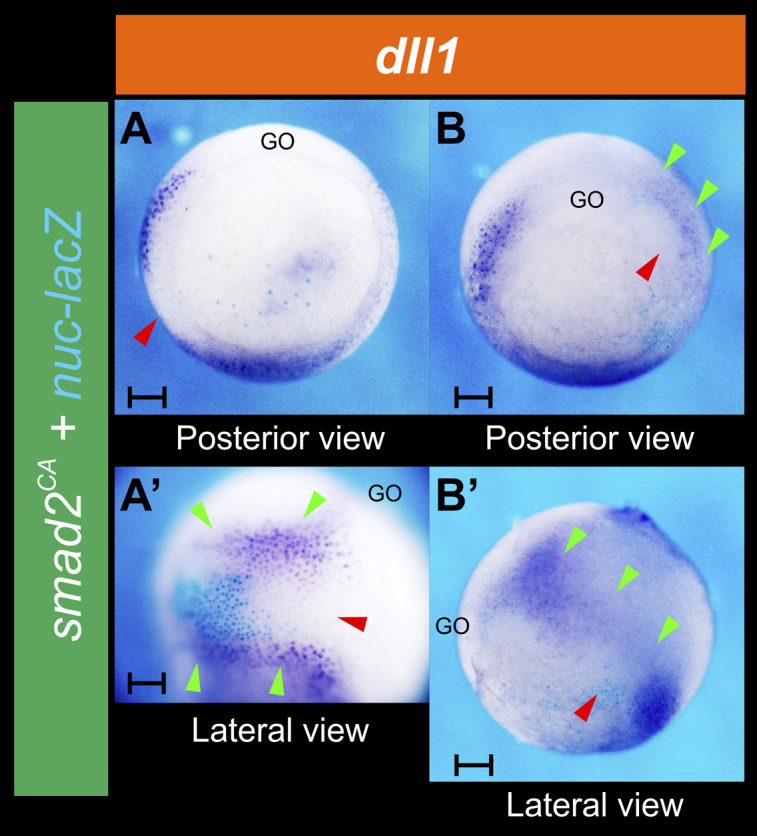
Effect of *smad2*^*CA*^ mRNA co-injected with *nuc-lacZ* mRNA tracer on the expression of *dll1*. **(A, A’)** and **(B, B’)** Two different albino embryos photographed in posterior (A, B) and lateral views (A’, B’) showing *dll1* expression at gastrula stage, as revealed by in situ hybridization. They were injected into one cell at the four-cell stage with 1 ng of *smad2*^*CA*^ + 0.5 ng of *nuc-lacZ* mRNAs as tracer. The enzymatic β-galactosidase activity in descendants of the injected cell was revealed with Xgal (turquoise staining). The red arrowheads indicate *dll1* repression; green arrowheads, ectopic induction of *dll1* at a distance from the *smad2*^*CA*^/*nuc*-*lacZ* expressing cells. The combined cell-autonomous repression with the ectopic induction of *dll1* by *smad2*^*CA*^ resulted in a shift of the *dll1* IMZ domain towards the ectoderm. GO, gastrula organizer. Scale bar: 0.2 mm.

*Dll1* is normally expressed in the IMZ, but it is turned off once cells have involuted (Wittenberger et al, 1999; López et al, 2005). Injection of *nuc-lacZ* mRNA or control MO did not affect *dll1* expression in the IMZ (Figs S1F–G’ and 3D, D’, and R). Gain of function with either *churc1VP16* or *churc1* mRNA significantly suppressed *dll1* in the IMZ (Figs 2J, O, and O’ and 3Q, Q’, and R). On the other hand, both, *churc1EnR* and *churc1* MO significantly expanded the *dll1* domain (Figs 2E, O, and O’ and 3H, H’, and R). The effect of *churc1* MO on *dll1* was specific since it was significantly rescued by *churc1* mRNA (Fig 3L, L’, and R). Our results demonstrate that, normally, *dll1* expression is inhibited in the territories where *churc1* is active and is thus restricted to the IMZ.

### In silico analysis shows that both *Xenopus laevis zeb2* homeologs contain putative Churc1 binding sites

An in vitro DNA binding selection assay (SELEX assay) previously determined that the chicken CHURC1 protein binds to an NGGGNN motif, with N representing any nucleotide with the frequencies shown in Fig S2A. Gel mobility shift and competition assays confirmed that CHURC1 specifically binds to this sequence (Sheng et al, 2003). In the same study, an in silico analysis of a 4,020 bp sequence of the human *ZEB2* gene, which included the promoter region, contained a significantly higher number of CHURC1 binding motifs than those expected by chance (Sheng et al, 2003).

Based on the evidence reported from the in silico analysis of the human *ZEB2* gene (Sheng et al, 2003), we performed a similar analysis for both *X. laevis zeb2* homeologs, focusing on those genomic regions comparable to the human *ZEB2* 4,020 bp region analyzed by Sheng et al (2003). As in human *ZEB2*, both regions of *zeb2.S* and *zeb2.L* included a predicted transcription initiation site, the first exon, the first intron, the second exon with the translational initiation site (ATG), and a similar sequence length.

When we compared the number of putative Churc1 binding motifs, we found that the *X. laevis zeb2* biological sequences contain 117 and 144 motifs for the *zeb2.S* and *zeb2.L* homeologs, respectively. In contrast, for the 10,000 simulated random *zeb2.S* sequences, we found an average of $\bar{x}$ = 65.45 ± 9.63 motifs per sequence, significantly less than the 117 motifs observed in the biological *zeb2.S* sequence (*P* < 0.00001; one sample Z-test). Similarly, for the 10,000 simulated random *zeb2.L* sequences we found an average of $\bar{x}$ = 70.47 ± 9.98 motifs per sequence, significantly less than the 144 motifs observed in the biological *zeb2.L* sequence (*P* < 0.00001, one sample Z-test) (Fig S2B). Additionally, an enrichment test was conducted with the MotifCounter R package (Kopp, 2017) and the result was a 2.68-fold enrichment for *zeb2.L* (*P* = 1.46 × 10^−7^) and a 1.92-fold enrichment for *zeb2.S* (*P* = 2.23 × 10^−3^) compared with their simulated random sequences.

These results show that coincidently with the findings shown for the human *ZEB2* gene (Sheng et al, 2003), both *X. laevis zeb2* homeologs contain a statistically nonrandom high number of putative Churc1 binding motifs in their genomic regions of interest analyzed here.

Coincident with the analysis of the human *ZEB2* gene addressing the quality of the NGGGNN sites (Sheng et al, 2003), the Logarithmic Likelihood (Log L) Sum scores for the *zeb2.S* and *zeb2.L* biological regions were significantly higher compared to the Log L sum of their corresponding simulated collections (not shown). However, this was indeed expected, since the collections of simulated sequences contain significantly lower numbers of NGGGNN sites (as demonstrated above, Fig S2B). Therefore, we performed an additional analysis of LogL distribution, for which we pooled all NGGGNN motifs present in the 10,000 simulated sequences. Then, we randomly selected from the pool the same number of NGGGNN motifs present in each biological *zeb2* region analyzed (117 y 144 for *zeb2.S* and *zeb2.L*, respectively). When we performed 1,000 non-parametric Kruskal-Wallis sum of ranks tests comparing the same number of motifs present in the biological sequences versus randomly selected motifs from the simulated sequences, no significant differences were obtained, but enrichment in motifs with LogL values higher than five can be observed in the biological sequences in comparison to the simulated sequences (Fig S2C). This supposes a distribution of motifs in the biological sequences with a higher probability of interacting with Churc1.

Next, we wondered if the motifs present in the biological sequences have a higher success rate to bind the Churc1 protein than the motifs present in the simulated sequences. To address this, we performed an A/B test comparing two β distributions. The “success rate” of binding Churc1 for the *zeb2.L* biological sequence has a 0.99876 probability of being higher than the “success rate” for the corresponding simulated sequences. For *zeb2.S*, this probability was 0.92322 (Fig S2D). Overall, we conclude that in terms of quality binding, for both *zeb2* homeologs it is very unlikely that the higher number of sites with a higher probability of binding Churc1 (according to the SELEX assay) in the biological *zeb2* sequences have arisen by chance.

Finally, we analyzed the local distribution of putative Churc1 binding motifs along each *zeb2* homeolog’s region of interest by scanning their sequences through a 150-nucleotides sliding window to evaluate if they are randomly distributed or if they are enriched in certain regions. We found regions with higher numbers of motifs than those expected by chance (Fig S2E). Therefore, putative Churc1 binding sites are not randomly distributed, but are enriched between the beginning of the first exon and the translational start site in both *zeb2.S* and *zeb2.L* regions analyzed. Similar results also were found in the same regions of the human *ZEB2* gene (Sheng et al, 2003).

In conclusion, the in silico analysis showed that coincidently with the human *ZEB2* gene, both *X. laevis* homeologs contain a high number of statistically not-random putative Churc1 binding sites near their predicted promoter regions, mostly concentrated upstream of the first and second exons, suggesting that *zeb2* might be a Churc1-direct target in *Xenopus*.

### Functional experiments demonstrate that *churc1* positively controls *zeb2*

**Figure 6.**
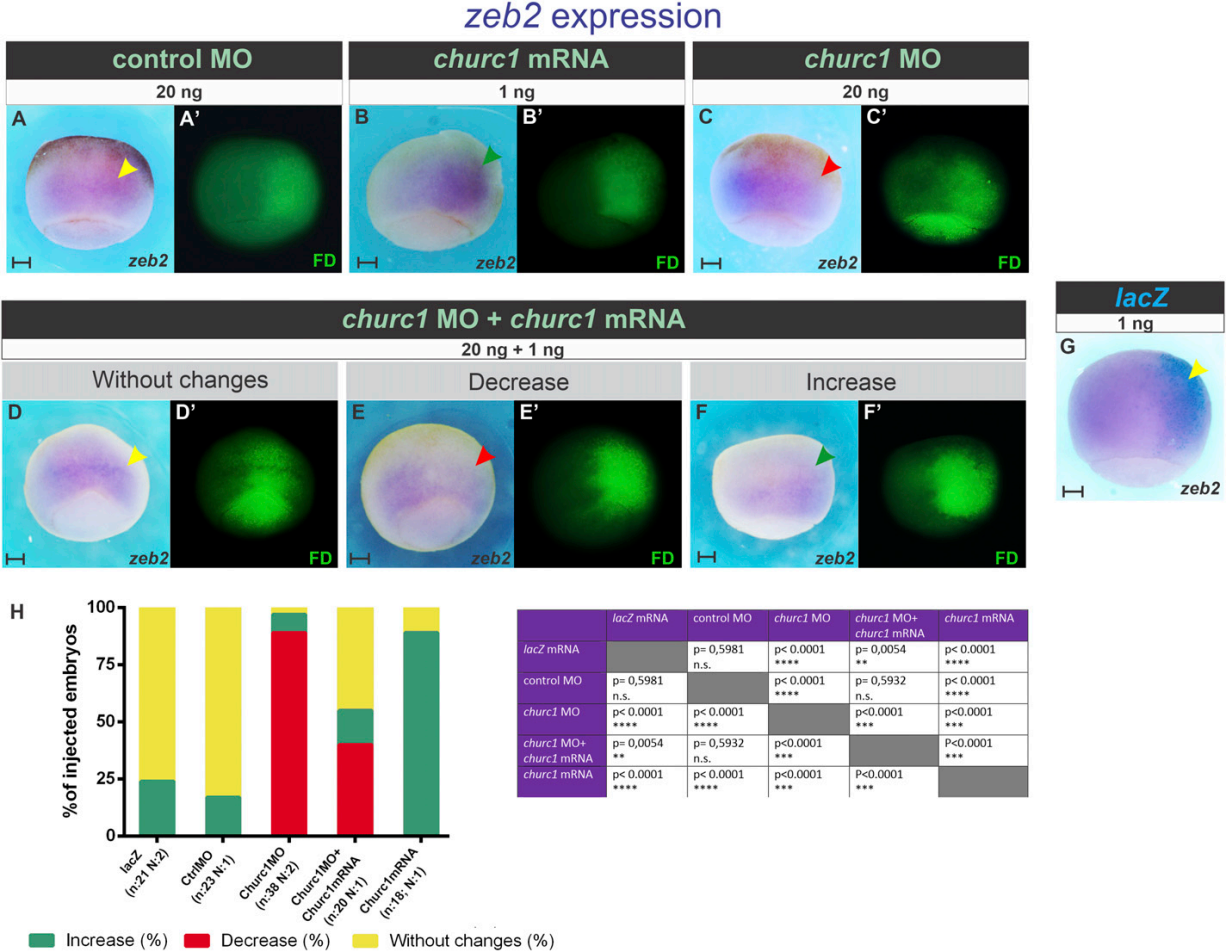
*churc1* positively controls *zeb2* expression in vivo during *Xenopus* gastrulation. **(A, B, C, D, E, F, G)** *zeb2* expression was analyzed by in situ hybridization at gastrula stage in embryos that were injected into one dorsal cell at the four-cell stage with the following molecules: (A) control MO (20 ng). **(B)** *churc1* mRNA (1 ng). **(C)** *church1* MO (20 ng). **(D, E, F)** *church1* MO (20 ng) + *churc1* mRNA (1 ng). **(G)** *nuc-lacZ* mRNA as control (1 ng). *zeb2* expression was compared between the injected- and the non-injected sides. **(A’, B’, C’, D’, E’, F’)** Fluorescence microscopy images corresponding to the bright field images shown in (A, B, C, D, E, F), respectively, revealing the green fluorescence of the dextran tracer (FD) which marks the injected side. **(G)** The injected side in (G) was revealed by the enzymatic activity of β-galactosidase (turquoise staining). In all photographs, the injected side is oriented towards the right. Red, green, and yellow arrowheads point to decreased, increased, or unperturbed *zeb2* expression on the injected side in comparison to the non-injected side. Scale bars: 0.2 mm. **(H)** Graphs comparing the effects on *zeb2* expression between *nuc-lacZ* mRNA (1 ng), control MO (20 ng), *churc1* MO (20 ng), *churc1* MO (20 ng) + *churc1* mRNA (1 ng), and *churc1* mRNA (1 ng). Graphs represent the percentages of injected embryos showing increase (green), decrease (red), or no changes (yellow) of *zeb2* expression on the injected side in comparison to the non-injected side. Asterisks indicate significant differences between treatments (*P* < 0.05; Chi-Square test). *P*-values are indicated in the table shown at the right. ns, non-significant differences. n, total number of analyzed embryos. N, number of independent experiments.

To corroborate if *zeb2* is regulated by *churc1* in vivo, we altered *churc1* function through overexpression and knockdown experiments and analyzed *zeb2* expression by ISH at gastrula stage. In nearly all embryos (16 of 18, 89% of injected embryos), *churc1* mRNA increased *zeb2* expression on the injected side (Fig 6B, B’, and H). These changes were statistically significant in comparison to embryos unilaterally injected with *nuc-lacZ* mRNA (Fig 6G and H). Knockdown with *churc1* MO produced the opposite result, decreasing *zeb2* expression in the great majority of embryos (34 of 38, 89% of injected embryos; Fig 6C, C’, and H), indicating that *churc1* is normally required for *zeb2* expression. This decrease was statistically significant in comparison to embryos unilaterally injected with control MO (Fig 6A, A’, and H) and was significantly rescued by co-injection of 1 ng of *churc1* mRNA (Fig 6D–H), confirming that the effects of *churc1* MO on *zeb2* were specific. We conclude that *churc1* positively controls *zeb2* in vivo during *Xenopus* gastrulation.

### *Dll1/Notch1* signaling positions the *hes4* domain in the NIMZ

**Figure 7.**
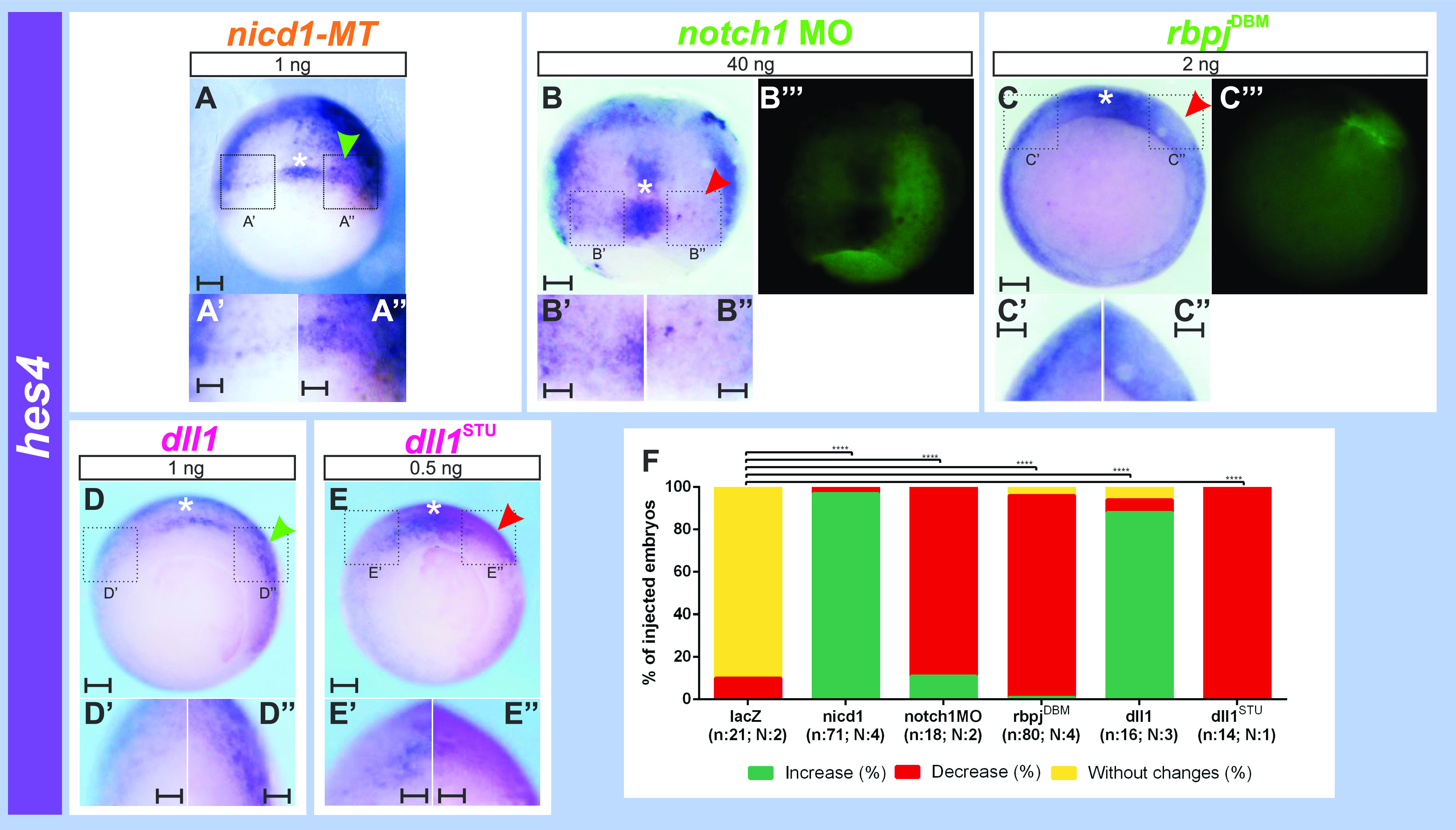
Dll1/Notch1 signaling positions the *hes4* domain demarcating the NIMZ. **(A, B, C, D, E)** *hes4* expression revealed by in situ hybridization at gastrula stage (NF11-11.5) in embryos that were unilaterally injected with: (A) 1 ng of *nicd1-MT* mRNA. **(B)** 40 ng of *notch1* MO. **(C)** 2 ng of *rbpj*^*DBM*^ mRNA. **(D)** 1 ng of *dll1* mRNA. **(E)** 0.5 ng of *dll1*^*STU*^ mRNA. Scale bars: 0.2 mm. **(A, B’’’, C’’’, D, E)** The injected side was revealed by MT immunolocalization (brown staining in A) or by the dextran tracer (green fluorescence in B’’’, C’’’; magenta staining in D, E). All photographs are oriented with the injected side towards the right. We evaluated the *hes4* stripes demarcating the NIMZ flanking *hes4* expression in the DML precursors (white asterisk). Red and green arrowheads point to decreased or increased *hes4* expression in the NIMZ domain, respectively, on the injected side in comparison to the non-injected side. **(A’, A’’, B’, B’’, C’, C’’, D’, D’’, E’, E’’)** Magnification of areas depicted within black dotted squares in (A, B, C, D, E), respectively. Scale bars: 0.1 mm. **(F)** Graph comparing the effects between 1 ng of *nicd1-MT* mRNA, 40 ng of *notch1* MO, 2 ng of *rbpj*^*DBM*^ mRNA, 1 ng of *dll1* mRNA, and 0.5 ng of *dll1*^*STU*^ mRNA on NIMZ *hes4* expression flanking the DML precursors. Graphs represent the percentage of injected embryos showing an increase (green), decrease (red), or no changes (yellow) in *hes4* expression, which was significantly increased by *nicd1* and *dll1* mRNAs, and significantly decreased (Chi-Square test; ****P = 0.0001) by *notch1* MO, *rbpj*^*DBM*^ mRNA, and *dll1*^*STU*^ mRNA in comparison to *lacZ* mRNA injections, which did not perturb *hes4* expression in this domain. 1.5 ng of *nuc-lacZ* or 2 ng of *cyt-lacZ* mRNAs were injected as control (see Fig S1E–E’’). n, total number of analyzed embryos; N, number of independent experiments.

To corroborate if *hes4* is regulated by *notch1/dll1* throughout the NIMZ, we analyzed *hes4* expression in this region, flanking the GO/DML precursors, in embryos in which we manipulated Dll1/Notch1 signaling. Activation of the Dll1/Notch1 pathway by overexpressing either the constitutively active Notch1 intracellular domain (NICD1) or the Dll1 ligand significantly expanded the NIMZ *hes4* domain (Fig 7A–A’’’, D–D’’, and F). In contrast, blocking Dll1/Notch1 signaling with *notch1* MO, a dominant negative construct of *rbpj* (*rbpj*^*DBM*^), or a dominant negative construct of *dll1* (*dll1*^*STU*^) decreased *hes4* expression in the NIMZ on the injected side (Fig 7B–B’’’, C–C’’’, E–E’’, and F) and this change was significant in comparison to *lacZ* mRNA injection (Figs S1E–E’’ and 7F). This confirms that Dll1/Notch1 signaling positively controls the *hes4* domain throughout the NIMZ. Since *dll1* is expressed in the IMZ and *hes4*, in the NIMZ, we propose that IMZ cells emit the Dll1 signal that activates *hes4* transcription in the neighboring NIMZ cells through the Notch1/Rbpj pathway.

### Nodal signaling positions the *dll1* and *hes4* domains in the MZ

Considering the key role of the Nodal pathway in the induction of germ layers and the role of the Notch pathway in their delimitation in vertebrates (Favarolo & López, 2018), we addressed if Nodal could control the Notch pathway in the MZ during gastrulation when germ layer segregation takes place. To this aim, we activated or blocked the Nodal pathway and analyzed *dll1* and *hes4* expression.

**Figure 8.**
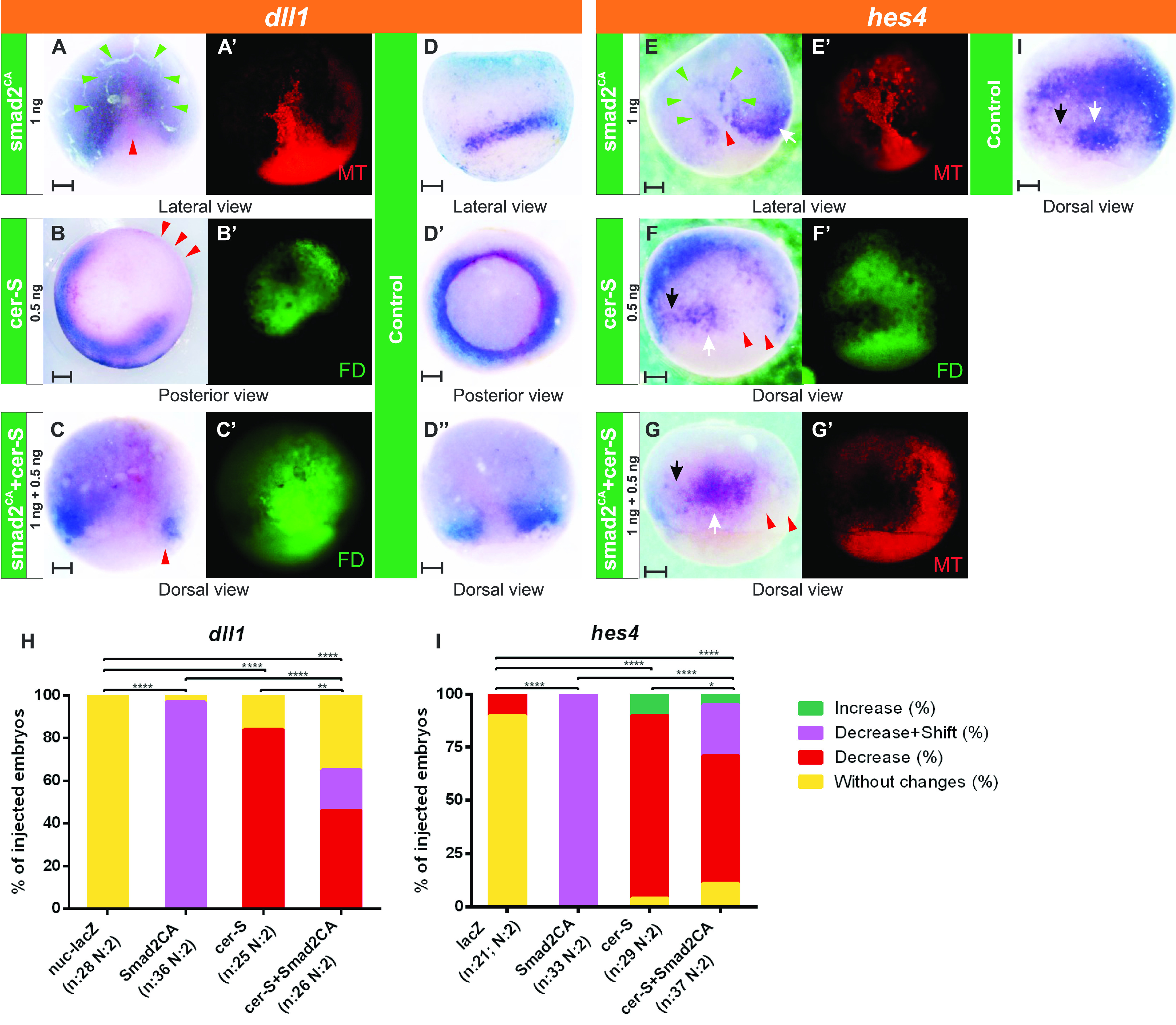
Effects of *smad2*^*CA*^ and *cer-S* on the expression of *dll1* and *hes4* in the MZ at gastrula stage. **(A, B, C, E, F, G)** Expression of *dll1* (A, B, C) and *hes4* (E, F, G) revealed by in situ hybridization at gastrula stage in embryos that were injected into one dorsal cell at the four-cell stage with: (A, E) 1 ng of *smad2*^*CA*^*-MT* mRNA. **(B, F)** 0.5 ng of *cer-S* mRNA. **(C, G)** 1 ng of *smad2*^*CA*^*-MT* mRNA + 0.5 ng of *cer-S* mRNA. **(D, D’, D’’)** *dll1* expression in uninjected sibling controls. **(I)** *hes4* expression in uninjected sibling controls. **(A’, B’, C’, E’, F’, G’)** Fluorescent images corresponding to the bright field views in (A, B, C, E, F, G), respectively, showing the injected side, as revealed by the c-Myc-tag epitope (MT, red immunofluorescence) **(A’, E’, G’)** or the FD tracer (green fluorescence) **(B’, C’, F’)**. In all photographs of injected embryos, the injected side is oriented towards the right, except for (A, A’) and (E, E’), showing lateral views. For all the injected embryos as those shown in (A, B, D, E, F, G), we compared the MZ expression of *dll1* (A, B, C) and *hes4* (E, F, G) between the injected- and the uninjected sides. For *hes4*, we evaluated the *hes4* stripes demarcating the NIMZ (black arrows) flanking *hes4* expression in the DML precursors (white arrows). Red arrowheads: repression; green arrowheads: ectopic induction at a distance from the *Smad2*^*CA*^ expressing cells. Cell-autonomous repression combined with ectopic induction of *dll1* and *hes4* by *smad2*^*CA*^ resulted in a shift of their domains towards the ectoderm. Scale bars: 0.2 mm. **(H, I)** Graphs comparing the effects on *dll1* (H) and *hes4* expression (I) of 1 ng of *nuc-lacZ* mRNAs as injection control (see Fig S1), 1 ng of *smad2*^*CA*^*-MT* mRNA, 0.5 ng of *cer-S* mRNA, and 1 ng of *smad2*^*CA*^*-MT* mRNA + 0.5 ng of *cer-S* mRNA. The bars represent the percentage of injected embryos showing decrease (red), decrease + shift (purple), increase (green), or no changes (yellow) in *dll1* (H) and *hes4* expression (I). n, total number of analyzed embryos; N, number of independent experiments. Chi-square test; ****P < 0.0001; **P = 0.0093; *P = 0.0156. Differences are considered as significant when *P* < 0.05.

For activation of the Nodal pathway, we injected *smad2*^*CA*^*-MT* mRNA, which encodes a constitutively active (CA) form of Smad2 (the Nodal pathway’s effector) fused to a Myc tag epitope (MT) (Müller et al, 2000). Strikingly, *dll1* expression was suppressed in cells with the highest levels of Smad2^CA^ protein, as revealed by immunofluorescence of the fused MT (Fig 8A, A’, and H) or by the β-Galactosidase activity resulting from the co-injected *nuc-lacZ* mRNA tracer (Fig S3), whereas it was ectopically activated in neighboring cells in the ectoderm (Figs 8A, A’, and H and S3). This cell-autonomous repression of *dll1* accompanied by a non-cell-autonomous induction of *dll1* resulted in a significant and dramatic shift of the *dll1* IMZ domain towards the ectoderm on the injected side (Figs 8A, A’, and H and S3; compare with uninjected control in Fig 8D). Injection of *nuc-lacZ* mRNA alone did not affect *dll1* expression (Figs S1 and 8H). A similar and significant shift was obtained for the Notch target gene *hes4* domain in the NIMZ after *smad2*^*CA*^*-MT* mRNA injection (Fig 8E, E’, and I). This evidence indicates that cells in which the Nodal cascade was strongly active emitted a signal that induced *dll1* at a distance and hence triggered the Notch/*hes4* pathway.

**Figure 9.**
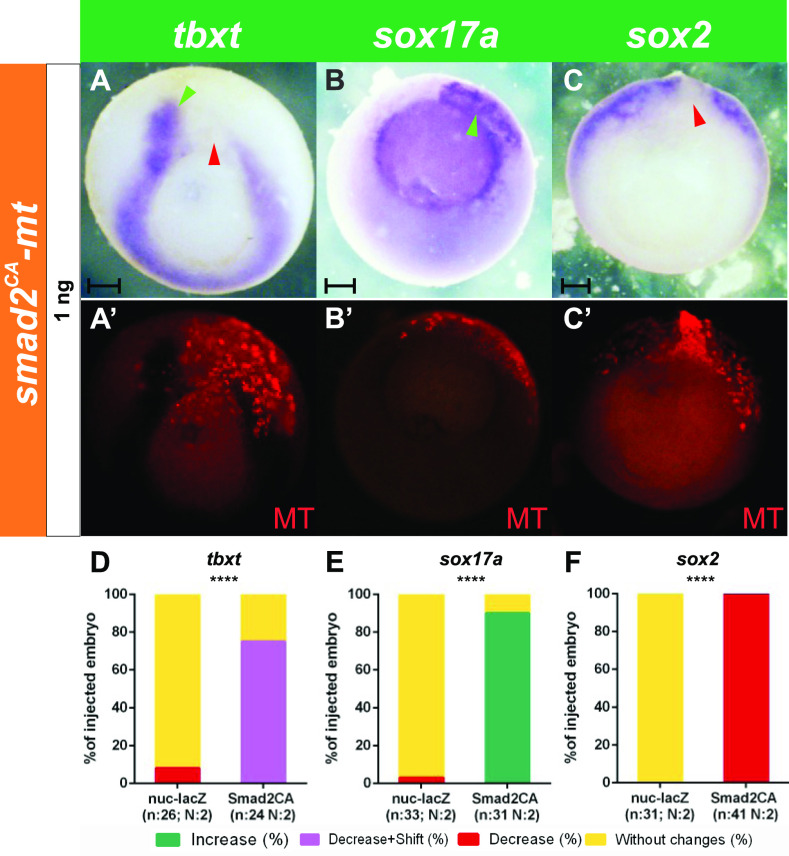
Effects of *smad2*^*CA*^ on germ layer specification markers at gastrula stage. **(A, B, C)** Expression of *tbxt* (A), *sox17a* (B), and *sox2* (C) revealed by in situ hybridization at gastrula stage in embryos that were injected into one dorsal cell at the four-cell stage with 1 ng of *smad2*^*CA*^*-MT* mRNA. **(A’, B’, C’)** Fluorescent images corresponding to the bright field views shown in (A, B, C), respectively, revealing the injected side by the MT immunofluorescence (red), corresponding to the MT epitope encoded by *smad2*^*CA*^*-MT* mRNA. The change in *tbxt*, *sox17a*, and *sox2* expression was evaluated by comparing the injected-with the contralateral uninjected side. **(A, A’)** *Smad2*^*CA*^ cell-autonomously repressed the mesoderm specification marker *tbxt* (red arrowhead) and induced it in neighboring cells (green arrowhead). **(B, B’)** *Smad2*^*CA*^ cell-autonomously induced the endoderm specification marker *sox17a* (green arrowhead). **(C, C’)** *Smad2*^*CA*^ repressed the neuroectoderm specification marker *sox2* (red arrowhead). Scale bars: 0.2 mm. **(D, E, F)** Graphs comparing the effects on *tbxt* (D), *sox17a* (E), and *sox2* expression (F) of 1 ng of *nuc-lacZ* mRNAs as injection control (see Fig S1) and 1 ng of *smad2*^*CA*^*-MT* mRNA. Results are expressed as the percentage of injected embryos showing decrease (red), decrease + shift (purple), increase (green), or no changes (yellow) for each marker; n; total number of analyzed embryos. N, number of independent experiments. The injection of *smad2*^*CA*^*-MT* mRNA produced significant changes in comparison to *nuc-lacZ* mRNA injections as control (Chi square test, ****P < 0.0001). The difference is considered as significant when *P* < 0.05.

To evaluate if this shift of *dll1* and *hes4* expression was correlated with a shift in the boundaries between germ layers, we analyzed the expression of their specification markers. *Smad2*^*CA*^ also significantly and cell-autonomously repressed the mesodermal marker *tbxt* but induced it in neighboring cells (Fig 9A, A’, and D). In contrast, the endodermal *sox17a* domain was significantly expanded, invading the mesoderm (Fig 9B, B’, and E), whereas the neural marker *sox2* was significantly suppressed (Fig 9C, C’, and F). Our results indicate that high levels of *smad2*^*CA*^ cell-autonomously induce endoderm, repressing the alternative, mesodermal fate, and concomitantly, suppressing *dll1* in the IMZ. These endodermal cells induced by *smad2*^*CA*^, in turn, release an intercellular signal that induces mesoderm and *dll1* expression in neighboring cells.

Cerberus is a secretion factor that normally binds to Nodal, BMP4, and Wnt proteins, inhibiting their activity. A truncated form, Cerberus-short (Cer-S), only retains the ability to bind Nodal (Piccolo et al, 1999). Thus, we injected *cer-S* mRNA, which has been successfully employed as a general Nodal antagonist to inhibit mesoderm and endoderm induction in plenty of works (Piccolo et al, 1999; Engleka et al, 2001; Kofron et al, 2004; Castro Colabianchi et al, 2021). On the injected side, *cer-S* suppressed *dll1* in the IMZ (Fig 8B, B’, and H; also compare with uninjected control in Fig 8D’) and *hes4* in the NIMZ (Fig 8F, F’, and I), demonstrating that Nodal is necessary for establishing their MZ domains.

Since *nodal5/6* induce *nodal1/2*/*4* (Takahashi et al, 2000), we wondered if the *smad2*^*CA*^-induced intercellular signal that activates *dll1* and *hes4* in neighboring territories involves Nodal. To test this hypothesis, we co-injected *smad2*^*CA*^ and *cer-S*. We found that, on the injected side, *cer-S* prevented the *smad2*^*CA*^-induced ectopic, non-cell-autonomous activation of *dll1* (Fig 8C, C’, and H; also compare with uninjected control in Fig 8D’’) and *hes4* (Fig 8G, G’, and I). This confirms that Nodal non-cell-autonomously mediates the induction of *dll1* and *hes4* produced by *smad2*^*CA*^.

Overall, our results demonstrate that Nodal signaling positions the MZ expression domains of a gene encoding a Notch ligand (*dll1*) and a Notch-target gene (*hes4*) responsive to Dll1/Notch1 signaling in the MZ.

## Discussion

**Figure 10.**
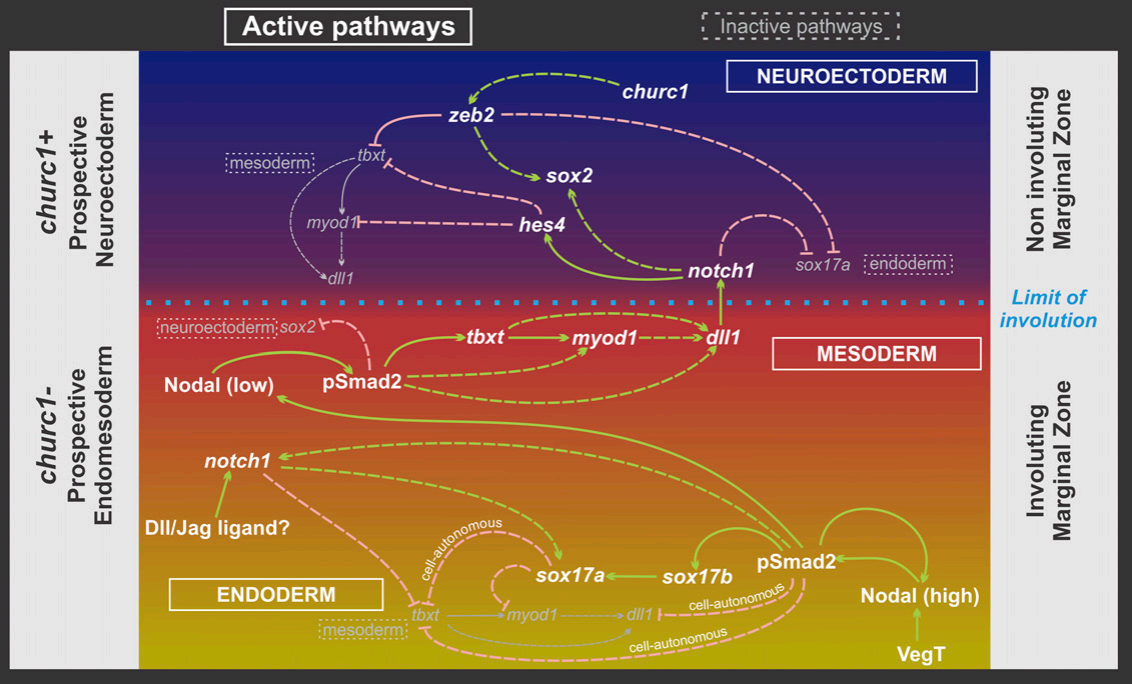
Model integrating the network involving Churc1, Nodal and Notch-dependent pathways in the induction and segregation of germ layers in the *Xenopus* MZ. During blastula stages, Nodal and Churc1 roughly outline neuroectoderm (blue) and endomesoderm (yellow/red) presumptive territories in the NIMZ and IMZ, respectively, contributing to establishing and restricting *dll1* expression to the IMZ during gastrulation. *dll1* is repressed in the NIMZ, where *churc1* is active, and induced through a relay Nodal cascade in the pre-involuted mesoderm in the IMZ, where *churc1* is inactive. High Nodal favors endoderm (yellow) over mesoderm specification (red). Endodermal cells, in turn, emit a lower wave of Nodal signaling, promoting mesoderm specification and *dll1* expression in the IMZ pre-involuted mesoderm. Dll1 signaling activates the Notch1 pathway on the NIMZ, which represses mesoderm specification through *hes4* and promotes neuroectoderm, thus refining the boundaries between them. Notch1 activity (perhaps triggered by another Dll/Jag ligand) contributes to endomesoderm segregation in the IMZ, favoring endoderm over mesoderm. White bold letters/thicker lines: regionally active pathways. Small gray letters/thinner gray lines: regionally inactive pathways. Green and pink lines represent positive and negative regulation, respectively. Full lines: direct regulation, with the strongest strength of connection according to experimental evidence. Broken lines: proposed regulation according to the available experimental evidence. See Table S1 summarizing the findings of the present work and experimental evidence from references supporting this model (Hopwood et al, 1989; Coffman et al, 1990; Frank & Harland, 1991; Harvey, 1991; Smith et al, 1991; Essex et al, 1993; von Dassow et al, 1993; Gurdon et al, 1994, 1995; Jones et al, 1995; Hudson et al, 1997; Joseph & Melton, 1997; Mizuseki et al, 1998; Steinbach et al, 1998; Clements et al, 1999, 2003; Kofron et al, 1999; Osada & Wright, 1999; Remacle et al, 1999; Verschueren et al, 1999; Watanabe & Whitman, 1999; Wittenberger et al, 1999; Yasuo & Lemaire, 1999; Agius et al, 2000; Eisaki et al, 2000; Lerchner et al, 2000; Osada et al, 2000; Takahashi et al, 2000; Weber et al, 2000; Davis et al, 2001; Hill, 2001; Engleka et al, 2001; Howell et al, 2002; Papin et al, 2002; Schohl & Fagotto, 2002; Yang et al, 2002; López et al, 2003, 2005; Sheng et al, 2003; Tsuji et al, 2003; Abe et al, 2004; Nitta et al, 2004, 2007; Sinner et al, 2004, 2006; Cui, 2005; Yamaguti et al, 2005; Steiner et al, 2006; Zamparini et al, 2006; Howard et al, 2007; van Grunsven et al, 2007; Cao et al, 2008, 2012; Miazga & McLaughlin, 2009; Luxardi et al, 2010; Revinski et al, 2010; Sakano et al, 2010; Kinoshita et al, 2011; Rousso et al, 2011; Skirkanich et al, 2011; Matsukawa et al, 2012; Aguirre et al, 2013; Bates et al, 2013; Gentsch et al, 2013; Chiu et al, 2014; Vega-López et al, 2015; Wills & Baker, 2015; Reid et al, 2016; Session et al, 2016; Charney et al, 2017a; 2017b; Karimi et al, 2018).

The *Xenopus* MZ is a transition area between germ layers where their limits are defined during gastrulation. We show that Nodal signaling and the Churc1 cascade, operating in the developing endomesoderm and neuroectoderm, respectively, position a Notch signaling territory in the MZ. A Dll1 domain is set on the IMZ, from where it activates *hes4* expression in the NIMZ through the Notch1 receptor. This Notch signaling territory, in turn, ensures mesoderm and neuroectoderm delimitation, thus refining germ layer segregation. The model integrating Churc1, Nodal, and Notch pathways in the induction and segregation of germ layers is presented in Fig 10. Below, we discuss our results and the evidence collected from other works supporting this model (for the detailed experimental evidence, see Table S1).

**Figure S4.**
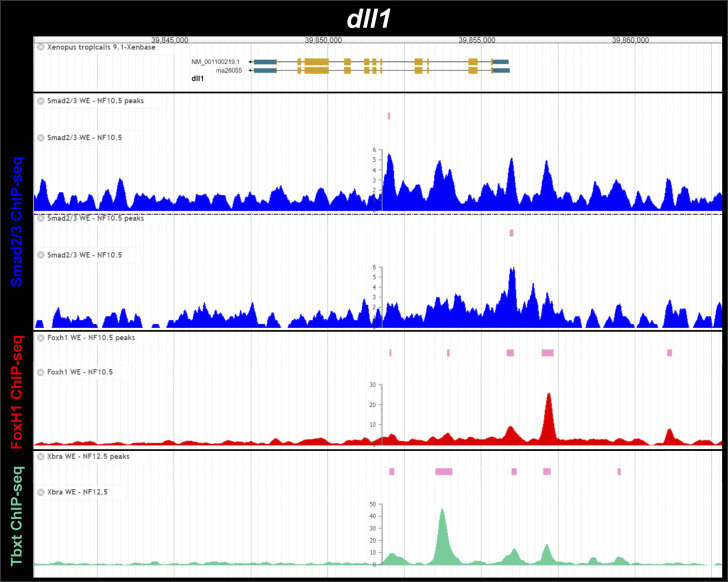
Smad2/3, Foxh1 and Tbxt (Xbra) binding near the *Xenopus tropicalis dll1* gene during gastrulation. ChIP-Seq analysis data of NF10.5 embryos and NF11-12.5 embryos from Chiu et al (2014) and Gentsch et al (2013). Snapshots were obtained from Xenbase (Karimi et al, 2018) (http://www.xenbase.org/, RRID:SCR_003280) and show the gene structure (gene model, upper track), the tracks of ChIP-peaks calls for Smad2/3, FoxH1 and Tbxt binding (pink boxes), and the tracks of their corresponding ChIP-seq profiles (blue, red, and green, respectively). Two biological replicates are shown for Smad2/3 ChIP-seq binding.

**Figure S5.**
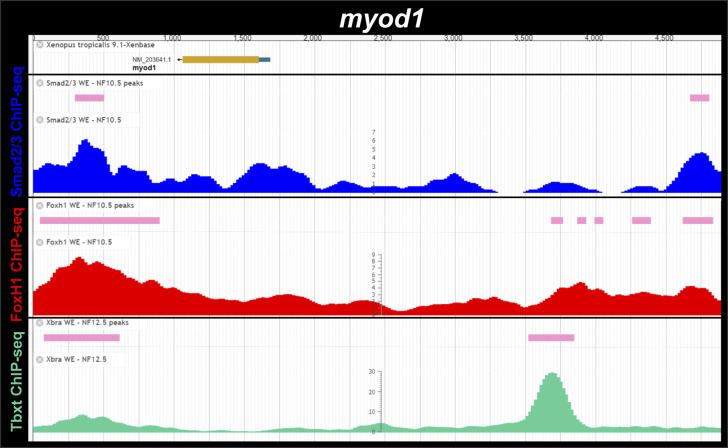
Smad2/3, Foxh1 and Tbxt (Xbra) binding near the *Xenopus tropicalis myod1* gene during gastrulation. ChIP-Seq analysis data of NF10.5 embryos and NF11-12.5 embryos from Chiu et al (2014) and Gentsch et al (2013). Snapshots were obtained from Xenbase (Karimi et al, 2018) (http://www.xenbase.org/, RRID:SCR_003280) and show the gene structure (upper track), the tracks of ChIP-peaks calls for Smad2/3, FoxH1 and Tbxt binding (pink boxes), and the tracks of their corresponding ChIP-seq profiles (blue, red, and green, respectively). The *myod1* locus corresponds to an assembled scaffold and the gene model is still uncomplete.

**Figure S6.**
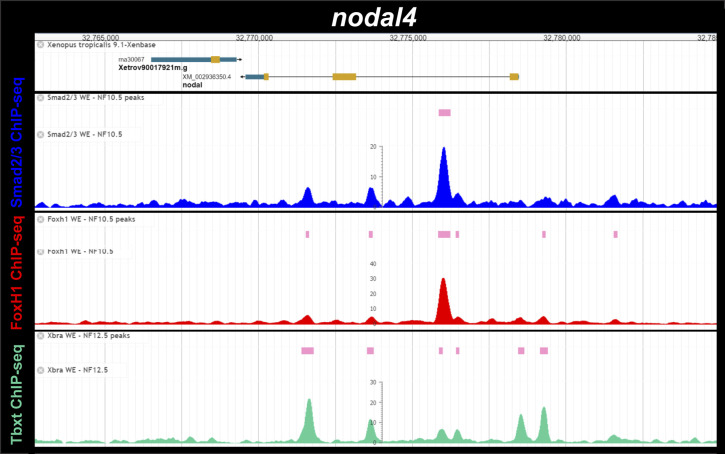
Smad2/3, Foxh1 and Tbxt (Xbra) binding near the *Xenopus tropicalis nodal4* gene during gastrulation. ChIP-Seq analysis data of NF10.5 embryos and NF11-12.5 embryos from Chiu et al (2014) and Gentsch et al (2013). Snapshots were obtained from Xenbase (Karimi et al, 2018) (http://www.xenbase.org/, RRID:SCR_003280) and show the gene structure (gene model, upper track), the tracks of ChIP-peaks calls for Smad2/3, FoxH1 and Tbxt binding (pink boxes), and the tracks of their corresponding ChIP-seq profiles (blue, red, and green, respectively).

**Figure S7.**
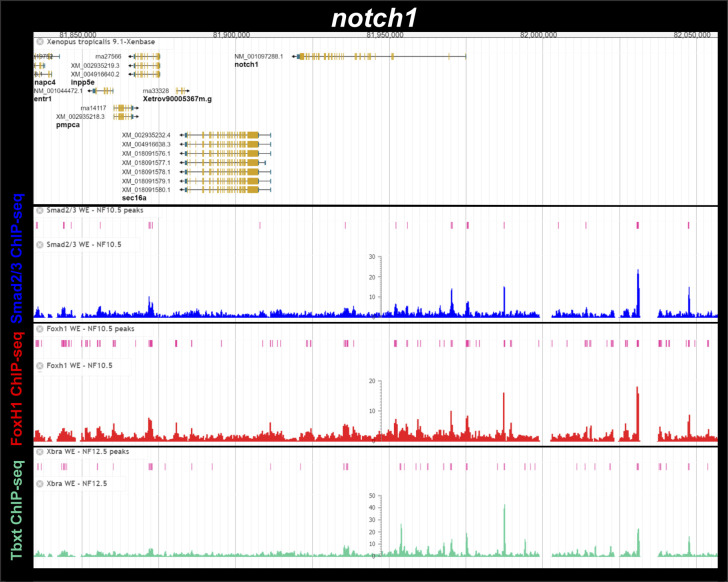
Smad2/3, Foxh1 and Tbxt (Xbra) binding near the *Xenopus tropicalis notch1* gene during gastrulation. ChIP-Seq analysis data of NF10.5 embryos and NF11-12.5 embryos from Chiu et al (2014) and Gentsch et al (2013). Snapshots were obtained from Xenbase (Karimi et al, 2018) (http://www.xenbase.org/, RRID:SCR_003280) and show the gene structure (gene model, upper track), the tracks of ChIP-peaks calls for Smad2/3, FoxH1 and Tbxt binding (pink boxes), and the tracks of their corresponding ChIP-seq profiles (blue, red, and green, respectively).

**Figure S8.**
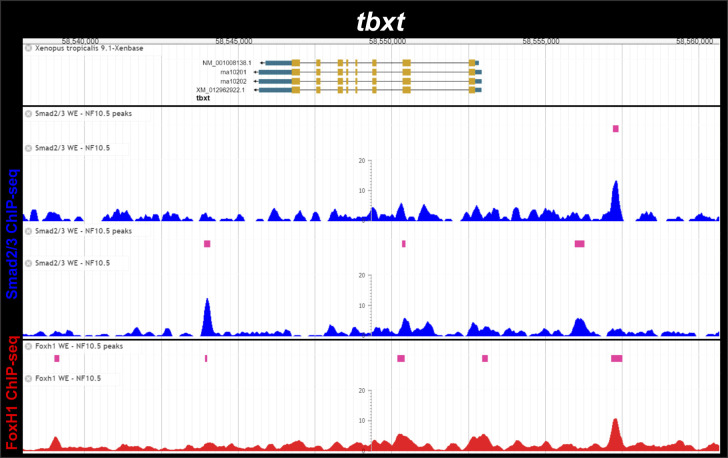
Smad2/3 and Foxh1 binding near the *Xenopus tropicalis tbxt* (*Xbra*) gene during gastrulation. ChIP-Seq analysis data of NF10.5 embryos from Chiu et al (2014). Snapshots were obtained from Xenbase (Karimi et al, 2018) (http://www.xenbase.org/, RRID:SCR_003280) and show the gene structure (upper track), the tracks of ChIP-peaks calls for Smad2/3 and FoxH1 (pink boxes), and the tracks of their corresponding ChIP-seq profiles (blue and red, respectively). Two biological replicates are shown for Smad2/3 ChIP-seq binding.

Table S1 Supporting experimental evidence for Fig 10. Connections between signaling pathways (TGFβ/Nodal; Notch), genes encoding transcription factors (*churc1*, *myod1*, *sox17*, *tbxt*, *zeb2*), and genes directly, indirectly, or putatively regulated by them (“target genes” proposed in Fig 10) are listed in separate tabs. Headings for pathways are colored in yellow; for transcription factors, in green, and for “target genes,” in blue. The “Strength of Connection” column summarizes the availability (+) of binding evidence and regulation evidence (gain of function, loss of function, dominant-negative, regulation in the absence of protein synthesis) described in the corresponding columns. In some cases, under the column “binding evidence,” we provide ChIP-Seq analysis data obtained from different sources, which is presented in Figs S4–S8. All evidence assembled in this table was obtained from *Xenopus* embryos, except for some cases related to *churc1*, which are indicated. All references cited in this table are listed in the References tab and in the Reference list of the main text. Evidence from the present work is shown in green letters. RNAseq data from Session et al (2016): expression levels at intermediate stages between eggs and NF8 were not analyzed in this study. CHX+ indicates an immediate response of the gene to the signaling pathway tested, without mediation of protein synthesis; CHX-indicates a non-immediate response of the gene to the signaling pathway tested, requiring mediation of protein synthesis. Abbreviations: CHX, cycloheximide (inhibitor of protein synthesis); D, dorsal; DBM, DNA binding mutant; Dex, dexamethasone; *dll1*^*STU*^, antimorphic form of *Xenopus laevis* Dll1, lacking the intracellular domain; DML, dorsal midline; DMZ, dorsal marginal zone; dn, dominant-negative; DNIMZ, dorsal non-involuting marginal zone; DSL, Delta/Serrate/Lag2 family of Notch ligands; EnR constructs, protein of interest fused with the repressor domain from *Drosophila* Engrailed protein; FP, floor plate; GR-constructs, hormone-inducible forms of proteins with nuclear functions under the control of the ligand binding domain of the human glucocorticoid receptor; hpf, hours post-fertilization; HH, Hamburger Hamilton stages for chicken development; IF, immunofluorescence; IMZ, involuting marginal zone; ISH, in situ hybridization; MBT, mid-blastula transition; MO, antisense morpholino oligonucleotide; NF, Nieuwkoop and Faber stages for *Xenopus* development; *nicd1*, construct encoding the intracellular domain of Notch1 (constitutively active form); NIMZ, non-involuting marginal zone; P-Smad2, phosphorylated Smad2 (active form of Smad2); *rbpj*^*DBM*^, DNA binding mutant form of *X. laevis* Rbpj that binds to NICD but lacks the ability to bind target sites in the DNA, behaving as a dominant-negative protein by forming non-functional complexes; *smad2*^*CA*^, construct encoding a constitutively active form of Smad2; TLE, transducin-like enhancer of split; V, ventral; VMZ, ventral marginal zone; VP16 construct, protein of interest fused to the activation domain of the Herpes simplex virus VP16 protein.

### Role of *churc1* in *Xenopus* germ layer development

In this work, we found that *Xenopus churc1* expression already is detected at mid-blastula, when a massive wave of zygotic transcription begins (Collart et al, 2014). *churc1* mRNA is present in the dorsal ectoderm at the onset of neural induction, which begins at the blastula BCNE center (Kuroda et al, 2004). Expression is restricted to the presumptive neuroectoderm, persisting in the *sox2* territory at early gastrula and developing neural plate. Although the avian *CHURC1* pattern was not reported in pregastrula embryos, *Xenopus* (this work) and chicken orthologs (Sheng et al, 2003) are similarly expressed from the beginning of gastrulation. In *Xenopus*, *churc1* transcripts are present in cells undergoing neural induction but absent from the involuting endomesodermal lineage (this work). This pattern is consistent with a role in neuroectoderm development in frogs, as previously proposed for birds, where *CHURC1* prevents the activation of key mesodermal genes and blocks cell ingression through the primitive streak by activating *ZEB2* (Sheng et al, 2003). In *Xenopus*, *zeb2* encodes a transcriptional repressor expressed in the dorsal ectoderm fated to become the neural plate (Eisaki et al, 2000; van Grunsven et al, 2000; Papin et al, 2002). Here, we show that in *Xenopus*, *churc1* expression overlaps the *zeb2* domain and that both *zeb2* homeologs contain putative Churc1 binding sites near their predicted promoters (this work), resembling their distribution in the human *ZEB2* gene (Sheng et al, 2003). Moreover, through overexpression, knockdown, and rescue experiments, we show that *churc1* positively regulates *zeb2* in vivo and controls germ layer development during *Xenopus* gastrulation.

It was reported that *churc1* overexpression prevented *tbxt* induction by FGF in the *Xenopus* animal cap assay and that both, *churc1* and *churc1VP16*, but not *churc1EnR*, suppressed *tbxt* in *Xenopus* and chicken embryos, suggesting a transcriptional activator role for Churc1 that indirectly represses *tbxt* (Sheng et al, 2003). While these authors reported that *churc1EnR* could not repress *tbxt*, we show that it strongly expanded the *Xenopus tbxt* domain, which invaded the territory normally occupied by the neuroectoderm. By extending the analysis to other germ layer markers and performing knockdown experiments, we demonstrate that normally, *churc1* not only inhibits mesoderm specification but also disfavors endoderm development whilst favors neuroectodermal fates in *Xenopus*, since *sox2* expression was suppressed with *churc1EnR* or reduced with *churc1* MO, and this was accompanied by a complementary expansion of the involuting lineages.

According to the most recent version of the *X. laevis* genome (v10.1), *churc1* is present as a singleton (*churc1.S*, Xenbase XB-GENEPAGE-853239). Therefore, it is unlikely that the milder effects of *churc1* MO compared with the *churc1EnR* construct are due to compensation by another homeolog. It is also unlikely that *churc1EnR* produces stronger, off-target effects than *churc1* MO because of competition with other endogenous Churc1-related proteins. Eukaryotic Churchill proteins form a unique family characterized by a zinc-binding region not shared with other zinc finger domain proteins (NCBI CDD pfam06573) and there appear to be no paralogues in eukaryotes for the only member of this family. On the other hand, it is not uncommon that milder effects are obtained with morpholinos than with dominant-negative constructs with potent transcriptional regulation domains. Indeed, milder effects of *churc1* MO than those obtained with a *churc1EnR* construct were also observed in zebrafish embryos (Londin et al, 2007). *churc1EnR* was designed to potently repress all Churc1-target genes, whereas knockdown with *churc1* MO probably does not completely prevent *churc1* translation. In theory, a higher dose of *churc1* MO might result in a stronger translational inhibition of endogenous *churc1* mRNA, but in our hands, injection of 40 ng of *churc1* MO resulted in high lethality, making impossible the analysis of germ layer phenotypes. Therefore, all analyses were done with 20 ng. The target sequence for the *churc1* MO employed in this study spans 23 nucleotides of the 5′UTR sequence, just upstream of the ATG translational start site. Although with different strengths, the effects of *churc1* MO and *churc1EnR* were similar, and *church1* MO effects were rescued by co-injection of *churc1* mRNA lacking the 5′ UTR. All this evidence indicates that the effects of blocking *churc1* function either with *churc1EnR* or with *churc1* MO were specific.

In conclusion, *Xenopus churc1* normally controls the limit of involution by exerting opposite functions on neuroectoderm and endomesoderm development. *churc1* is expressed in the non-involuting lineages where it restricts endomesoderm and favors neuroectoderm development, while its absence from the IMZ lineage allows endomesoderm development (Fig 10).

In chick embryos, *CHURC1* was proposed to foster epiblast competence to respond to neural inducers (Sheng et al, 2003). This was based on the observation that, when electroporated at Hamburger-Hamilton stage 4 (HH4, definitive primitive streak) in the chick epiblast of the area opaca (which is competent to respond to neural inducers from an ectopic GO until HH4, but normally does not contribute to neural tissue), *CHURC1* could sensitize these cells to activate SOX2 expression after implanting an ectopic GO at HH5 stage (Sheng et al, 2003). However, electroporation at intermediate primitive streak stages (HH3) with *CHURC1VP16* did not affect *SOX2* expression, despite strongly repressing the mesodermal markers *TBXT* and *TBX6* (Sheng et al, 2003). Here we show that *churc1* mRNA could increase *sox2* expression at gastrula stage in *Xenopus*, as long as the expression of the gene encoding the neural inducer Chrd.1 was not severely abolished. However, unlike our injections at the four-cell stage in *Xenopus*, electroporation of *CHURC1VP16* DNA in chick embryos was performed after the onset of gastrulation. These differences in experimental conditions may account for the dissimilarities in the results of *sox2* expression between species, where neural induction is thought to begin before gastrulation (Kuroda et al, 2004; Stern, 2005, 2006). Indeed, the gene encoding the neural inducer CHRD is already expressed at pregastrula stages in the precursors of the GO in chick embryos (Streit et al, 1998) as well as its ortholog *chrd.1 *is expressed at pregastrula stages in the BCNE in *Xenopus* (Kuroda et al, 2004). Therefore, activating the *CHURC1* cascade after the onset of gastrulation might be too late to induce *SOX2* expression, as shown in chicken (Sheng et al, 2003).

In this work, we also show that the activating Churc1 form suppressed *dll1* expression, whereas *churc1EnR* and *churc1* MO expanded the *dll1* domain over the NIMZ. Therefore, *churc1* prevents *dll1* expression in the NIMZ, restricting it to the IMZ. Since *churc1* is already expressed at mid-blastula (this work), thus preceding the onset of *dll1* expression in the IMZ at early gastrula (López et al, 2005), our evidence indicates that *churc1* is upstream of the Dll1/Notch1 cascade previously proposed to refine the limit of involution during germ layer segregation (Revinski et al, 2010).

In addition, previous findings (summarized in Table S1 and Fig 10) support that the transcriptional repressor Zeb2 might be mediating the role of Churc1 in promoting neural fate and preventing the activation of *tbxt*, *dll1*, and *sox17* (endomesoderm program) in the *Xenopus* NIMZ. *Xenopus zeb2* overexpression neuralized animal caps without inducing mesoderm (Eisaki et al, 2000). Mouse Zeb2 binds the *X. tbxt* promoter in vitro and represses *X. tbxt* in vivo at gastrula stage (Verschueren et al, 1999). ZEB2 binding sites in the *X. tbxt* promoter are necessary to restrict *tbxt* expression to the IMZ, since they prevent ectodermal ectopic expression (Remacle et al, 1999; Lerchner et al, 2000). Mouse *Zeb2* cell-autonomously repressed *tbxt* in *Xenopus* in an immediate-early way. Conversely, a dominant negative *Xenopus* Zeb2 (fused to the VP16 transactivating domain) activated *tbxt* and *sox17a* in animal caps, in an immediate-early way (Papin et al, 2002). However, *Xenopus zeb2* knockdown did not affect *tbxt* expression, although it did block neural development (Nitta et al, 2004). These authors suggested that other members of the Zeb2 family of transcription factors might cooperate in repressing *tbxt* expression outside the IMZ.

### Nodal positions a Notch signaling territory in the MZ

In this work, we show that constitutively active Smad2^CA^ cell-autonomously induced the endodermal marker *sox17a* and repressed *tbxt*, *dll1*, and *hes4*. In addition, cells at a distance from those expressing the highest levels of Smad^CA^ ectopically activated *tbxt*, *dll1*, and *hes4*. This non-cell autonomous induction was mediated by Nodal secreted by those cells expressing the highest levels of Smad2^CA^. In addition, there is plenty of evidence that *tbxt* is repressed by high- and induced by low levels of Nodal signaling, whereas *sox17a* is induced by high levels of Nodal and represses *tbxt* cell-autonomously (Gurdon et al, 1994, 1995; Latinkić et al, 1997; Yasuo & Lemaire, 1999; Weber et al, 2000; Engleka et al, 2001) (Table S1 and Fig 10). Therefore, the model we present in Fig 10 contemplates that Nodal signaling, initially activated in the vegetal hemisphere by VegT (see the Introduction section), induces Smad2 phosphorylation (pSmad2, the active form of Smad2) in the endomesoderm. In those cells receiving the highest levels of Nodal signaling, Smad2 activity is strongest, promoting endoderm specification and cell-autonomously repressing mesoderm specification and *dll1*, probably through *sox17* (Table S1). In turn, these endodermal cells signal to their neighbors through a second wave of Nodal, which in lower doses induces mesoderm specification, *tbxt*, and *dll1* expression. In the IMZ, *tbxt* positively contributes to the activation of *dll1* in the presumptive pre-involuted mesoderm, possibly directly and through the activation of *myod1* (Table S1). Interestingly, Zeb2 binds to the sequence 5′-CACCT, which overlaps with the E2 box sequence 5′-CACCTG, which is recognized by some bHLH transcription factors like Myod1. This suggests that Zeb2 might be repressing target genes by competing with positive regulators like Myod1 (Verschueren et al, 1999).

The sequential Nodal activation cascade described above contributes to endoderm and mesoderm segregation from the endomesoderm, ensuring that *dll1* is expressed in the presumptive, pre-involuted mesoderm. In turn, from the pre-involuted mesoderm in the IMZ, Dll1 activates the Notch1 cascade on the NIMZ, promoting *sox2* expression and neuroectoderm specification, while repressing *tbxt* and mesoderm specification through *hes4*. Interestingly, two homeodomain binding sites at the *tbxt* regulatory region are necessary to repress this gene in the ectoderm at mid-gastrula. Between them, there is a putative Rbpj binding site of unknown significance (Lerchner et al, 2000), suggesting that *tbxt* might be directly controlled by Notch/Rbpj signaling.

### Concluding remarks

*churc1* transcripts are present in the presumptive neuroectoderm at mid-blastula transition and persist during gastrulation in *Xenopus*. *churc1* favors neuroectoderm over endomesoderm development, positively regulates *zeb2*, and prevents the expression of the gene encoding the Notch ligand Dll1 in the neuroectoderm. *churc1* is not expressed in the IMZ, thus relieving *tbxt*, *dll1*, and *sox17a* to be transcribed in this region.

On the other hand, through a relay cascade, Nodal signaling prevents *dll1* expression in the endoderm but induces it in the presumptive mesoderm. Thus, Nodal signaling controls the position of the MZ stripe of Dll1/Notch activity alongside endomesoderm induction and segregation between endoderm and mesoderm.

Once the mesoderm was induced by Nodal signaling and *churc1* has delineated the presumptive neuroectoderm territory, the activation of *dll1* in the IMZ refines the boundaries between mesoderm and neuroectoderm through the *notch1*/*hes4* cascade. In addition, we have previously proposed that Notch1 signaling also contributes to endomesoderm segregation and this might be triggered by another Dll/Jag ligand, distinct from Dll1 (Table S1 and Fig 10) (Revinski et al, 2010).

## Materials and Methods

### Embryological manipulations, RNA synthesis, morpholinos, and injections

Albino and wild-type *X. laevis* embryos were obtained using standard methods by natural mating or by in vitro fertilization (Sive et al, 2010) from adult animals obtained from Nasco, and staged according to Nieuwkoop and Faber (1994). Protocols were approved by the Laboratory Animal Welfare and Research Committee (CICUAL) from Facultad de Medicina, Universidad de Buenos Aires.

Synthetic capped mRNAs for microinjection were obtained as follows. Plasmids *smad2*^*CA*^*-Myc-tag* (*MT*) (in pCS2+MT) (gift from Uwe Strähle) (Müller et al, 2000), *cer-S* (in pSC2+) (gift from Eddy de Robertis) (Bouwmeester et al, 1996), *nicd1-MT* (in pCS2+MT) (Chitnis et al, 1995) (gift from Chris Kintner), *cyt-lacZ* (in pCS2+), *nuc-lacZ* (in pCS2+) (gifts from David Turner) (Turner & Weintraub, 1994) were digested with NotI; *churc1* (in pSC2+) (gift from Claudio Stern) (Sheng et al, 2003) was digested with SacII. They were in vitro transcribed with the mMESSAGE mMACHINE Sp6 Kit (AM1340; Ambion). Plasmids *churc1EnR* (in pUT-EnR MT) and *churc1Vp16* (in pUT-VP16) (gift from Claudio Stern) (Sheng et al, 2003) were digested with EcoRI and in vitro transcribed with the T7 Megascript transcription kit (AM1334; Ambion) with a 4:1 cap analog:GTP ratio, using m7G(5′)ppp(5′)G (AM8050; Ambion) and T3 Megascript transcription kit (AM1330; Ambion) with a 4:1 cap analog:GTP ratio, using m7G(5′)ppp(5′)G (AM8050; Ambion), respectively. Capped mRNAs were purified with the RNeasy mini kit (74104; QIAGEN).

Translation blocking antisense morpholino oligonucleotides (MO) were used for *churc1* (5′-GTCGCGCTCCTAACTACGGATAC-3′) and *notch1* (5′-GCACAGCCAGCCCTATCCGATCCAT-3′) (Gene Tools). The *notch1* MO was previously used and validated in works by our group and by other authors (López et al, 2003; Revinski et al, 2010; Sakano et al, 2010; Acosta et al, 2011; Castro Colabianchi et al, 2018). As control morpholino (control MO), we used the standard control oligo or the random control oligo 25-N (Gene Tools).

Injections were delivered into the animal hemisphere at ∼30–40° from the equator of one dorsal cell at the four-cell stage in wild-type embryos or in one cell at the two-cell stage in albino embryos. The injected amounts of synthetic mRNAs and morpholinos are indicated in the figures. The injections included molecular tracers such as 40 ng of Dextran Oregon Green 488, MW 10000, anionic lysine fixable (DOG, D7171; Thermo Fisher Scientific), 40 ng of Dextran, Fluorescein, 10,000 MW, anionic, lysine fixable (FD, D1820; Thermo Fisher Scientific), or 20 ng of Dextran, Biotin, 10,000 MW, lysine fixable (BDA-10000, D1956; Thermo Fisher Scientific). The injected side was detected by revealing the distribution of the co-injected tracer, as previously described (Revinski et al, 2010); of β-galactosidase activity for the *lacZ* constructs, as previously described (Franco et al, 1999), or of the Myc-tag epitope (MT) encoded by the injected mRNA, as described below.

### ISH and immunodetection

Plasmids for obtaining antisense RNA probes for whole-mount ISH were linearized with the appropriate restriction enzyme and in vitro transcribed with the appropriate RNA polymerase as follows: *dll1*, with XhoI/T7 (gift from Eric Bellefroid) (Chitnis et al, 1995); *not*, with HindIII/T7 (*pBS-KS-Xnot* plasmid, gift from David Kimelman) (von Dassow et al, 1993); *sox2*, with EcoRI/T7 (*pBS-sox2* plasmid, gift from Yoshiki Sasai) (Kishi et al, 2000); *tbxt*, with SalI/SP6 (*αbra-pSP64T* plasmid, gift from Abraham Fainsod) (Smith et al, 1991); *sox17a*, with SmaI/T7 (*pBS-SK-sox17a* plasmid, gift from Hugh Woodland) (Hudson et al, 1997); *hes4*, with BamHI/T7 (*pBS-SK+-hes4* plasmid, gift from Dave Turner) (Turner & Weintraub, 1994); *churc1*, with BamHI/T7 (*pSC2+churc1* plasmid, gift from Claudio Stern); *zeb2*, with PstI/T3 (*pCRscript.Xsip1* plasmid, gift from James C. Smith) (van Grunsven et al, 2000).

The preparation of digoxigenin-labeled antisense RNA probes and the whole-mount ISH procedure were performed as previously described (Pizard et al, 2004), except that the proteinase K step was omitted. We were not able to detect *churc1* transcripts by our standard ISH conditions, perhaps explaining why the expression pattern was still unavailable. However, with slight modifications to our protocol, by lowering 5°C the hybridization temperature and prolonging the alkaline phosphatase (AP) reaction for 24 h in an alkaline phosphatase buffer with suboptimum pH to prevent background staining (Kinoshita et al, 2006), we could visualize a distinct expression pattern. Therefore, for *churc1*, the ISH procedure included the following modifications: the prehybridization, hybridization, and SSC washing steps were performed at 55°C instead of 60°C; 0.2×, 1×, and 2× SSC concentrations were tested for the washing steps and gave similar results; the digoxigenin-AP labeled probe was revealed using a pH:7.5 AP buffer (100 mM Tris pH 7.5, 50 mM MgCl_2_, 100 mM NaCl, 5 mM levamisole) (Kinoshita et al, 2006). After ISH, pigmented embryos were bleached as previously described (Acosta et al, 2011).

Embryos injected with *smad2*^*CA*^*-mt* mRNA were fixed overnight at 4°C with MEMPFA, transferred to 100% ethanol, and kept at −20°C until being processed for ISH. After the ISH procedure, embryos were processed for immunodetection of the MT epitope as follows. They were rehydrated in 50% methanol in MAB (100 mM maleic acid, 150 mM NaCl pH7.5), washed with MAB, and incubated for 5 min at room temperature in blocking buffer (2% Blocking Reagent; Roche, Cat. no. 11 096 176 001, prepared in MAB). Then, embryos were incubated overnight at 4°C with the primary antibody (anti-c-Myc IgG1, mouse monoclonal antibody; Hybridoma Bank, 910E) diluted 1/200 in blocking buffer, washed five times, 60 min each with MAB, and incubated overnight at 4°C in the dark with the secondary antibody (anti mouse IgG+IgM [H+L] Alexa-594 [Jackson 115-585-044]) diluted 1/200 in blocking buffer. Then, the antibody was washed five times, 60 min each, with MAB and transferred to 1× PBS for visualization and image acquisition. The MT epitope encoded by the *nicd1-mt* construct was detected by immunohistochemistry as previously described (López et al, 2005). Embryos were photographed in an MVX10 fluorescence microscope (Olympus) equipped with a DP72 camera (Olympus).

### Bioinformatic analysis

*X. laevis* is one of many allotetraploid species derived from the fusion of two diploid ancestor species. Therefore, it contains two different subgenomes, named L (for long) and S (for short) because their chromosomes differ in length. After allotetraploidization, the orthologs derived from the diploid L and S ancestors became L and S “homeologs” in allotetraploid species. In *X. laevis*, around 56% of the homeolog pairs (as in the case of *zeb2*), were retained, whereas other homeologs from the L or S subgenomes were lost. The remaining ones are present as “singletons” (as in the case of *churc1*) (Kondo & Taira, 2022).

For sequence retrieval and characterization of both *X. laevis zeb2* homeologs, the *zeb2.S* and *zeb2.L* open reading frame and upstream intergenic sequences were downloaded from Xenbase (https://www.xenbase.org/). The transcription initiation sites were predicted by Promoter 2.0 Prediction Server (Knudsen, 1999). Then, a subsequence of 4,340 and 4,662 bp upstream from the predicted transcription initiation site, for *zeb2.S* and *zeb2.L* respectively, were further analyzed.

For background (bg) sequence generation, nucleotide compositions for both *X. laevis zeb2* homeologs’ regions of interest were obtained with DNA Stats through the Bioinformatics.org server (Stothard, 2000). Then, 10,000 simulated random sequences with the same length and nucleotide composition as the *zeb2.S* and *zeb2.L* regions of interest were generated with the RANDNA tool (Piva & Principato, 2006).

For both biological and simulated sequences, the DNA Pattern Find tool (Stothard, 2000) was used to find all motifs matching the Position Weight Matrix (PWM) derived from the in vitro DNA binding selection assay (SELEX assay) previously determined for chicken CHURC1 protein (Sheng et al, 2003). After motif quantification, a one-sample Z-test was conducted using the distributions3 R-package version 0.1.2 (R Team, 2017). The sequence logos were generated using the MotifStack R package v1.34 (Ou et al, 2018).

For the Likelihood analysis of Churc1 binding, first, we calculated the probability of each motif in every background (bg) sequence for *zeb2.S* and *zeb2.L*, i.e., P(NGGGNN_i_|bg *zeb2.S*) and P(NGGGNN_i_|bg *zeb2.L*) according to the nucleotide composition used to generate the bg sequences. Then, we calculated the probabilities of each site for both simulated and biological sequences using the PWM from Sheng et al (2003), i.e., P(NGGGNN_i_|SELEX Churc1 motif). Finally, the Log-likelihood for each site was calculated as LogL (NGGGNN_i_) = Log [(P(NGGGNN_i_)|Selex Churc1 motif)/(P(NGGGNN_i_)|bg)]. To evaluate the distribution of Churc1 binding Log likelihoods in biological and background sequences, first, all motifs present in the 10,000 simulated sequences were pooled. Then, we randomly selected from the pool the same number of motifs present in each biological *zeb2* region. A non-parametric Kruskal-Wallis sum of ranks test was performed. Finally, we replicated the test 1,000 times with a new random selection of simulated Churc1 binding motifs from the pool each time.

A Shapiro-Wilk normality test was conducted to evaluate the normality of the distribution of LogL from the SELEX experiment (Log L_SELEX_). Next, we assumed as being “successful” those motifs with a Log L higher than the mean Log L_SELEX_ value, and as a “failure” those motifs with a Log L lower than the mean Log L_SELEX_ value. Then, through Monte Carlo simulations (MonteCarlo R package v1.09) (Hallgren, 2013), we compared the distribution of “successful” motifs of the biological regions against the simulated sequences to do an A/B test comparing two β distributions (100,000 events simulation). We used an a priori β distribution of 0.35.

The local enrichment of putative Churc1 binding sites was analyzed using a 150-nucleotides sliding window to study the distribution of putative Churc1 binding site motifs along each *zeb2* homeolog region of interest. The global enrichment was evaluated using the motifcounter R package v 1.20.0 (Kopp, 2017). For the calculation of the *P*-value, a compound Poisson approximation was used.

### Data collection and statistics

Sibling embryos were randomly allocated to control or experimental groups and fixed when untreated control siblings reached the desired stage. For each injected embryo, the ISH expression domain of the analyzed marker was compared between the injected side and the contralateral non-injected side. Results are expressed as a percentage of the total number (n) of embryos with the indicated phenotypes, which are described in the main text or the figure legends. The number of biological replicates (N) analyzed is indicated for each set of experiments in the figures. Biological replicates represent batches of embryos from independent mating pairs. For statistical analysis of ISH results, a Chi-square test was applied using the GraphPad Software and the results are included in the figures. Differences were considered significant when *P* < 0.05. The main text details statistical analysis for in silico assays of the *X. laevis zeb2* sequences.

## Supplementary Material

Reviewer comments
